# Neurobehavioral Effects of Two Distinct Anti-Inflammatory Drugs in a Two-Week Chronic Stress Model in Zebrafish

**DOI:** 10.3390/brainsci16080815

**Published:** 2026-07-31

**Authors:** Maria M. Kotova, Tatiana O. Kolesnikova, Sahil V. Amikishiev, Viacheslav D. Riga, Murilo S. de Abreu, Pavel E. Musienko, Allan V. Kalueff

**Affiliations:** 1 Neuroscience Department, Sirius University of Science and Technology, 354340 Sochi, Russia; kotova.mm@talantiuspeh.ru (M.M.K.);; 2 Department of Functional Biochemistry of the Nervous System, Institute of Higher Nervous Activity and Neurophysiology RAS, 117485 Moscow, Russia; 3 Graduate Program in Health Sciences, Federal University of Health Sciences of Porto Alegre, Porto Alegre 90050-170, Brazil; 4 Moscow Center for Advanced Studies, 123592 Moscow, Russia; 5 COBRAIN Center, M. Heratsi Yerevan State Medical University, Yerevan 0025, Armenia

**Keywords:** zebrafish, affective behavior, chronic unpredictable stress, neuroinflammation, neuroglia

## Abstract

**Background/Objectives**: Chronic stress and neuroinflammation (promoted by micro- and astroglia) represent key factors in affective pathogenesis. Complementing rodent models, zebrafish (*Danio rerio*) are widely used in experimental modeling of affective disorders. Here, we examined the effects of chronic treatment with two distinct anti-inflammatory drugs, minocycline and nimesulide, on behavior and neuroinflammation-related biomarker genes in the zebrafish chronic unpredictable stress (CUS) model. **Methods**: Following two-week CUS with or without drug treatment, fish were assessed using the novel tank test for anxiety-like behavior, and the zebrafish tail immobilization (ZTI) test for depression-like behavior. **Results:** A 14-day treatment with minocycline (50 mg/L) reduced general motor activity and brain expression of both pro-inflammatory (*nos2a*) and anti-inflammatory (*arg1*) microglial biomarker genes, as well as upregulated the anti-inflammatory astrocytic marker *s100a10*. In CUS-exposed fish, this treatment attenuated depression-like ZTI immobility behavior and downregulated both pro-inflammatory (*nos2a*, *cox2*) and anti-inflammatory (*arg1*, *ptx*) glial biomarkers compared to the stress-only control. In contrast, one-week nimesulide treatment during CUS produced an anxiolytic-like effect and normalized the expression of stress-induced pro-inflammatory glial biomarkers *cox2* and *il1β*. **Conclusions:** Overall, our findings suggest that the two anti-inflammatory drugs tested modulate behavioral and molecular responses to chronic stress in zebrafish, with minocycline demonstrating broader anti-neuroinflammatory and antidepressant-like effects, and nimesulide exhibiting more specific anxiolytic-like properties in this model.

## 1. Introduction

Chronic stress is a key factor in affective pathogenesis, including anxiety and depression [1,2,3]—the two most prevalent and highly comorbid brain disorders with severe clinical outcomes [4,5,6,7] and treatment resistance [8]. Mounting clinical evidence implicates inflammation in affective disorders [9,10,11,12], as depressed patients exhibit elevated peripheral levels of pro-inflammatory cytokines (e.g., interleukins 1-beta (IL-1β), 6 (IL-6), interferon gamma (IFN-γ), and tumor necrosis factor (TNF)) [13]. Elevated neuroinflammation is also consistently reported both clinically [14,15,16,17] and in animal models of affective states [18,19,20]. For instance, chronic unpredictable stress (CUS) in mice promotes pro-inflammatory cytokine production in the prefrontal cortex and upregulates neuroinflammation markers, such as TNF and cyclooxygenase (COX) 1 [21]. In rats, prolonged 12-week CUS elevates IL-1β mRNA and protein levels, activates the NLR family pyrin domain containing 3 (NLRP3) inflammasome, and induces depression-like behavior [22]. Similarly, chronic social stress in rodents triggers depression-like behaviors and increases brain expression of protein convertase subtilisin/kexin type 5 (Pcsk5), a contributor to neuroinflammatory pathways [23].

However, neuroinflammation is not merely a consequence, but an important trigger of affective disorders [24], as shown by overt psychiatric side effects following pro-inflammatory therapy (e.g., triggering depression in patients receiving IFN-α) [25,26]. Central neuroinflammation is mediated by neuroglia undergoing morphological and functional ‘activation’, shifting towards microglial pro-inflammatory cytotoxic M1 state [27,28]), and astroglia pro-inflammatory (A1) phenotype [28] overlapping and cross-activating with microglia [29,30]. Given its key role in affective pathogenesis, suppressing neuroinflammation can be a logical and promising alternative therapeutic strategy. For example, the selective COX-2 inhibitor celecoxib is used as an adjunct to conventional antidepressant therapy [31], and exerts intrinsic antidepressant effects in patients with osteoarthritis [32,33]. A common antibiotic minocycline is another agent modulating neuroinflammation due to its microglia-inhibiting properties [34]. Various animal models, especially rodents, are a valuable tool to study affective pathogenesis experimentally [18,19,20]. For instance, in the mouse chronic mild stress models, minocycline evokes anxiolytic- and antidepressant-like effects linked to the inhibition of microglial activation [34,35]. Clinical studies further support the efficacy of minocycline as an adjunctive therapy for affective disorders (e.g., showing positive outcomes in patients with treatment-resistant depression) [36,37,38].

Complementing well-established rodent models of affective disorders [21,22,23], a small freshwater teleost fish, the zebrafish (*Danio rerio*), represents a prominent model organism in translational neuroscience [39,40,41]. Its key advantages include high fecundity, rapid development, small size, genetic tractability and homology, as well as low maintenance costs, making it highly suitable for large-scale behavioral and pharmacological studies [42]. A wide array of validated behavioral tests for zebrafish assesses anxiety- and depression-like states, enabling robust evaluation of stress-related phenotypes [43,44]. The high conservation of key neuroendocrine systems, including stress-related neurotransmitters, hormones and their receptors, further supports the translational relevance of zebrafish models for stress research [42,45]. Additionally, multiple validated stress paradigms have been adapted for zebrafish, including CUS [46,47,48,49]. Capitalizing on this useful and powerful in vivo model system, here we examine the effects of chronic treatment with common anti-inflammatory drugs minocycline and nimesulide, on affective behavior and neuroinflammatory markers in zebrafish CUS.

## 2. Materials and Methods

### 2.1. Animals and Housing

The study utilized adult (7–9 months old) wild-type short-fin zebrafish (~50:50 male:female ratio) obtained from a local commercial distributor (Axolotl Co., St. Petersburg, Russia). Prior to the experiments, the fish were kept for at least 3 weeks under standard conditions in groups of 30 fish per a 40-L plastic tank (28 height × 60 lengths × 40 widths, cm) filled with filtered water, feeding twice a day with standardized flake feed (Tetra GMBH, Osnabruck, Germany). Water in the home tanks was fully changed once a week, and the tanks were carefully cleaned with detergent and then thoroughly rinsed with running water. The water used underwent filtering with reverse osmosis and was kept in the same room for at least 5 days before use, with baking soda and sea salt added to maintain water pH at 7–7.8, water temperature maintained at 26 ± 2 °C, water conductivity at 800–1600 µS/cm, alkalinity at 50–75 ppm (mg/L), and general hardness at 3–8 °dH. Lighting in the holding room was maintained at 950–960 lux (as assessed by a AE0903 Luminometer; Open Science, Moscow, Russia) with a 14/10 h light/dark cycle, according to zebrafish care standards (lights on at 08:00 AM) [50,51]. All animals were experimentally naive prior to testing. Animal husbandry complied with national and institutional guidelines and regulations. The outbred population selection for the present study was based on population validity considerations and their relevance for the present study. Briefly, although genetically controlled models (e.g., inbred zebrafish strains) can be more reproducible and reliable systems for genetics research, modeling central nervous system (CNS) disorders, such as in the present study, involves ‘real’ human disorders affecting genetically heterogeneous populations. Thus, using outbred zebrafish strain (such as selected here) was deemed a more populationally valid and translationally relevant approach for the purpose of this study [52].

During the experimental procedures, each treatment group was housed in a single dedicated holding tank (n = 15–30 fish per tank depending on experiment), to ensure that all individuals within a group were exposed to the chronic stressors uniformly and simultaneously, thereby minimizing inter-tank variability in stress application. To mitigate potential tank-specific environmental variables, standardized daily water renewal was performed between 10.00 and 11.00 a.m. (except for the days of testing), whereas fish were assigned to treatment groups using sequential randomization to ensure balanced baseline characteristics across groups and sexes (approximately 1:1 male-to-female). Drug-treated water renewal was performed during such procedures. To obtain independent behavioral measurements, each fish was tested individually in a separate, clean apparatus.

A total of 180 fish were used across all three experiments. Overall mortality across these experiments was 6%, and involved 11 individuals from 90 CUS-exposed fish (12% in this group). The sample size for behavior testing (n = 15 per group) was based on statistical power analysis for a difference in mean values between groups of 10–15, SD—10, alpha—0.05 and power—0.8. The sample size (n = 8 per group) for gene expression studies was calculated based on statistical power analysis for a difference in mean values between groups of 10, SD—5, alpha—0.05 and power—0.8. For Experiment 1, an initial cohort of 30 fish per group was utilized to buffer against possible mortality or behavioral or procedural drop-outs. Final sample sizes varied due to the strictly pre-defined exclusion criteria, which included spontaneous mortality during the stress protocol, occasional behavioral test invalidations (e.g., fish jumping out of the apparatus), and technical outliers in molecular analyses (specifically, when the difference in threshold cycle (Ct) values between technical replicates exceeded 0.5 cycles). In total, 33 fish were excluded across all experiments due to these pre-defined criteria.

### 2.2. Chronic Unpredictable Stress (CUS)

**Figure 1:**
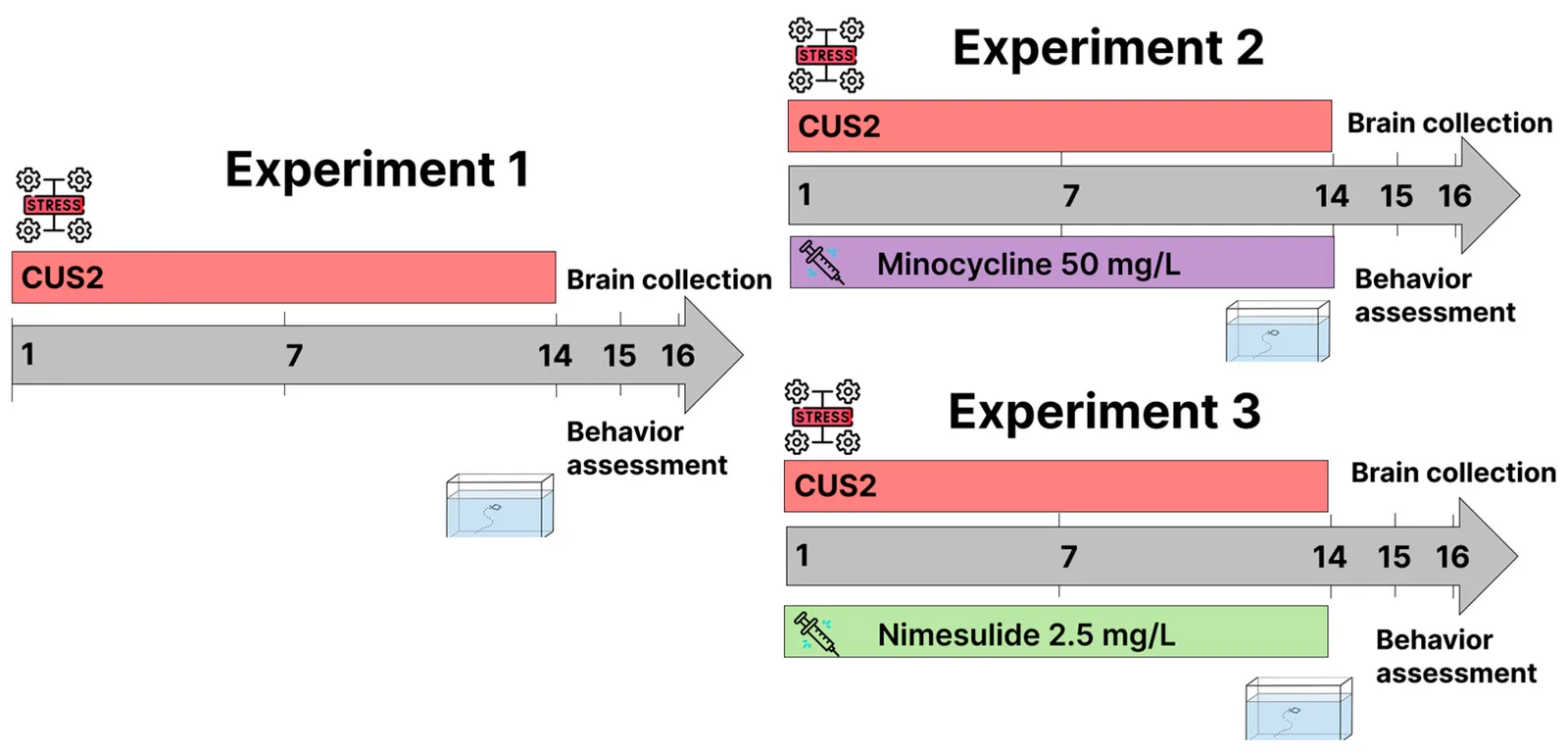
A general diagram summarizing the study experimental design. Briefly, Experiment 1 validated the effects of 2-week chronic unpredictable stress paradigm (CUS2) on anxiety- and despair-like behavior (n = 27 per group) and brain expression of selected neuroinflammation biomarker genes (n = 7 per group). Experiments 2 and 3 examined the effects of chronic administration of minocycline (50 mg/L for 2 weeks, n = 8–13 per group) and numesulide (2.5 mg/L for 1 weeks, n = 10–14 per group) during CUS2 on behavior and brain expression of selected neuroinflammation biomarker genes.

The study general experimental design and group details are shown in Figure 1 and Table 1. Experiment 1 examined the impact of a two-week CUS (CUS2) on zebrafish behavior and the expression of pro- and anti-inflammatory markers in neuroglia. During the CUS2 battery, the fish were kept in 5 L plastic home tanks (31 cm height × 17 cm length × 12 cm width) at the initial density of 4 fish/L, and subjected to various stressors daily for the 2 weeks, according to [53], with minor modifications (see Table 1 for details of CUS2 protocol used here). Control fish were maintained under identical housing conditions but not subjected to any stressors prior to testing and sampling.

The CUS2 protocol was selected for this study as it was a well-established paradigm in adult zebrafish, reliably inducing stress-related behavioral and molecular alterations [55] unlike a shorter, one-week paradigm that often fails to produce behavioral alterations [56]. Following the CUS2 exposure, zebrafish subgroups underwent behavioral assessment in the novel tank test (NTT, for anxiety-like and motor activity) and the zebrafish tail immobilization test (ZTI, for despair-like behavior) in Experiments 1–3. Euthanasia and sample collection were performed 24 h after the final behavioral test, to avoid immediate testing- and handling stress-associated confounding effects. Animals were sacrificed in ice water (followed by decapitation after the cessation of opercular movements for >30 s), their brains quickly dissected on ice, frozen in liquid nitrogen, stored at −80 °C and subsequently used to assess mRNA expression levels by PCR. In a separate pilot study, we also compared behavioral efficacy of minocycline vs. a common selective serotonin reuptake inhibitor (SSRI) fluoxetine (Supplementary Materials), chosen as a reference drug well-characterized in chronic stress research, including in fish models [53,57,58]).

### 2.3. Drug Administration

To probe putative effects of anti-inflammatory drugs on anxiety- and depression-like behavior in zebrafish CUS model, we used typical agents from two different pharmacological classes. A common tetracycline antibiotic, minocycline was selected for its well-established anti-stress effects due to the inhibition of microglial activation and suppression of microglia-mediated neuroinflammation [35]. A common non-steroidal anti-inflammatory drug (NSAID) nimesulide was selected here for its primary anti-inflammatory action via COX-2 inhibition, and its additional capacity to modulate astroglial inflammatory responses, as demonstrated in various in vitro models [59,60]. Drug concentrations were selected based on laboratory pilot experiments and the established literature. For minocycline (AVVA-RUS Ltd., Kirov, Russia), pilot studies evaluated 25, 50, and 100 mg/L. For nimesulide (pharmaceutical-grade granules for oral suspension, AVVA-RUS Ltd., Kirov, Russia), pilot studies evaluated 1, 2.5, and 5 mg/L.

Minocycline and nimesulide were administered in the present study via continuous water immersion. In Experiment 2, minocycline was administered at a concentration of 50 mg/L for two weeks, concurrent with the CUS2 protocol. This duration and concentration were chosen as the minimal effective dose to ensure robust modulation of neuroinflammation without inducing overt toxicity. In Experiment 3, nimesulide was administered at 2.5 mg/L for one week (i.e., during the second week of the CUS2 protocol); a shorter exposure period was chosen here since our pilot experiments revealed its toxicity and mortality when administered for 2 weeks of CUS2 (Supplementary Materials, Figure S2). Due to the inherent poor aqueous solubility of nimesulide, it was not dissolved as a true solution but was prepared as a homogeneous suspension in the system water. To ensure consistent exposure and prevent sedimentation, the suspension was thoroughly homogenized prior to, and during each daily water change. Because the target concentration of nimesulide was very low (2.5 mg/L), the amount of inactive ingredients added to the water from the granules was negligible and did not affect water quality or fish behavior.

Minocycline was diluted in dimethyl sulfoxide (DMSO, Paneco Ltd., Moscow, Russia) to ensure adequate solubility, and added to the home tanks. The final DMSO concentration was 0.02% (vol/vol), shown to be behaviorally inert in adult zebrafish [61]. Control groups for minocycline received system water containing 0.02% DMSO without the drug. Control groups for nimesulide received system water subjected to the identical daily handling and homogenization protocol. Throughout the exposure periods, fish were monitored daily for signs of overt toxicity. At the administered concentrations, no significant mortality, abnormal body condition, or severe disruption of feeding behavior was observed compared to the respective vehicle control groups. The duration of treatment, concentration, and route of fluoxetine administration in the additional experiment (Supplementary Materials Table S2 and Figure S3) were selected based on previous studies in zebrafish stress-related models [62].

### 2.4. The Novel Tank Test (NTT)

Behavioral testing was performed between 12:00 and 5:00 PM. Prior to behavioral testing, all fish were transported from the holding room and acclimated to the adjacent testing room (with similar temperature and lighting) for 2 h. After behavioral testing, fish were returned to their respective home tanks. The NTT for behavioral analyses was selected here as it is the most widely used behavioral assay in adult zebrafish [63,64]. The apparatus used was a custom-made acrylic rectangular tank (20 cm height × 20 cm length × 5 cm width) filled with system water (temperature 26 ± 2 °C) to a height of 19 cm, and divided virtually into two equal horizontal zones. Water parameters during testing were similar to those used for animal housing. 

During testing, the animals were gently removed from their holding tanks by a net and individually placed into the testing apparatus, where their behavior was recorded for 5 min by an SJ4000 action camera (SJCAM, Hong Kong, China), scoring distance traveled (cm), the number of top entries, time spent in top (s) and the latency to enter the top (s) of the tank, number and duration of periods of freezing (s) (immobility state), and duration of high mobility (s), based on the fish body center point. High mobility was defined here as duration of mobility periods greater than 60%, where mobility is the percentage of alteration in the area of the recorded animal relative to the previous time point. All behavioral analyses in this study were performed offline using the Noldus EthoVision XT17.5 software (Noldus IT, Wageningen, Netherlands).

### 2.5. The Zebrafish Tail Immobilization (ZTI) Test

The ZTI test was performed 1 h after the NTT. In this assay, the caudal half of the fish was fixed by a damp standard laboratory viscose sponge (7 cm length × 5.5 cm width × 4.8 cm height) cut in the middle to a depth of 2 cm from the bottom, as in [43] and attached to the top of a small transparent plastic beaker (7 × 5.5–4.8 cm) using two additional cuts on the sides. The cranial part of the fish body was left hanging freely in an upside-down position (vertically) in the beaker fully filled with system water, as in [43]. Fish were placed in the apparatus individually for 5 min, scoring total distance moved (cm), activity (%) and latency of the first activity period(s). The animals were individually placed in the apparatus and their behavior was recorded for 5 min by an SJ4000 camera and analyzed offline using the EthoVision XT17.5 software. Activity was calculated as the average number of pixels changed between each pair of consecutive video frames, relative to the pixel size of the entire setup arena and expressed as the mean percentage (%) of changed pixels per trial. Behaviors assessed in the present study fully adhered to the Zebrafish Neurobehavioral Catalog [65,66].

All experimenters were unaware of the treatment groups during behavioral testing and molecular assays, as well as during offline statistical and video analyses, using individual codes to identify fish/groups. Manual analyses of behavioral data were performed by two highly trained observers (blinded to the groups) with high inter- and intra-rater reliability of >0.85, as assessed by Spearman correlation as part of the laboratory’s standard operating procedure (SOP).

### 2.6. Brain mRNA Expression Level

Table 2 summarizes selected key biomarker genes used in the present study. The pro-inflammatory M1 and anti-inflammatory M2 microglial phenotypes were evaluated using the expression of *nos2a* (nitric oxide synthase 2a) and *cox2* (cyclooxygenase-2), and *arg1* (arginase-1) and *cd206* (mannose receptor C type 1), respectively, with *il1β* (interleukin 1β) and *il6* (interleukin 6) included as key pro-inflammatory cytokines secreted by M1 microglia [67,68]. The A1 and A2 astroglial phenotypes were assessed based on the expression of *c3* (complement component 3) and *gbp2* (guanylate-binding protein 2), and *s100a10* (s100 calcium binding protein a10) and *ptx* (pentraxin 3), respectively [69]. We also analyzed the expression of the glucocorticoid receptor gene (*gr*) as a genetic biomarker relevant to the impact of stress on the neuroendocrine axis.

The expression of selected genes was assessed in whole brain samples using two housekeeping genes (*β–act*/β—actin and *rpl13*/ribosomal protein l13) using the reverse transcription (RT-) PCR. RNA isolation was performed with an analog of Trizol ExtractRNA (Evrogen, Moscow, Russia, cat. no. SKBC032) according to the manufacturer’s instructions. The cDNA was next synthesized using oligo(dT)20 primers with equal amounts of RNA per sample (MMLV RT kit, Eurogen, Moscow, Russia, cat. no. SK006S/M) and the real time PCR procedure was performed using qPCRmix-HS SYBR kit (Eurogen, Moscow, Russia, cat. no. PK147L) and QuantStudio5 Amplifier (Thermo Fisher Scientific, Waltham, USA) in 3 replicates per sample. To ensure data reliability, technical replicates with a threshold cycle (Ct) difference > 0.5 cycles were excluded as outliers, consistent with our pre-defined quality control criteria. Gene expression levels were normalized to the expression level of the housekeeping genes using the ΔΔCT method, as in [56] (see Table 2 for the list of primers).

### 2.7. Statistical Analyses and Data Handling

Statistical analysis was performed using the GraphPad Prism 10 (GraphPad Software, San Diego, CA, USA). Data normality was assessed using the Shapiro–Wilk test. Given the non-parametric distribution of the data, primary 2-group comparisons were performed using the Mann–Whitney U-test. Additionally, to rigorously evaluate the 2 × 2 factorial design in Experiments 2 and 3 (with between-subjects factors Stress [present/absent] and Drug [present/absent]), a generalized linear model (GLM) was fitted, similar to [57]. The main effects of the two factors, together with their interaction, were evaluated by a Type III analysis of deviance (Wald χ^2^ tests, 1 df per term). Factors were effect-coded so that main-effect tests were averaged over the levels of the other factor. A significant Stress × Drug interaction was interpreted as the drug effect being conditional on the presence of stress. Following the identification of significant effects or interactions in the GLM, post hoc pairwise comparisons were conducted using non-parametric tests (Kruskal–Wallis test with post hoc Dunn’s correction) to determine specific group differences.

Statistical significance was defined as *p* < 0.05. Results for non-parametric analyses are presented as median and the interquartile range (IQR). To ensure maximum transparency, all relevant figures display individual data points alongside summary statistics. Outliers were identified and removed strictly using the ROUT method (Q = 1%) in GraphPad Prism to eliminate extreme technical or procedural anomalies. To ensure complete data transparency, particularly for endpoints with small sample sizes (e.g., RT-qPCR), we have provided a detailed breakdown of all data in the Supplementary Materials, including the results without outlier exclusion, alongside the specific distribution of the removed data points (Supplementary Materials Tables S3–S5, and Figures S4 and S5). Inter- and intra-rater reliability was assessed here using the Spearman correlation based on the NTT data.

## 3. Results

### 3.1. Behavioral and Transcriptional Effects of CUS2

**Figure 2:**
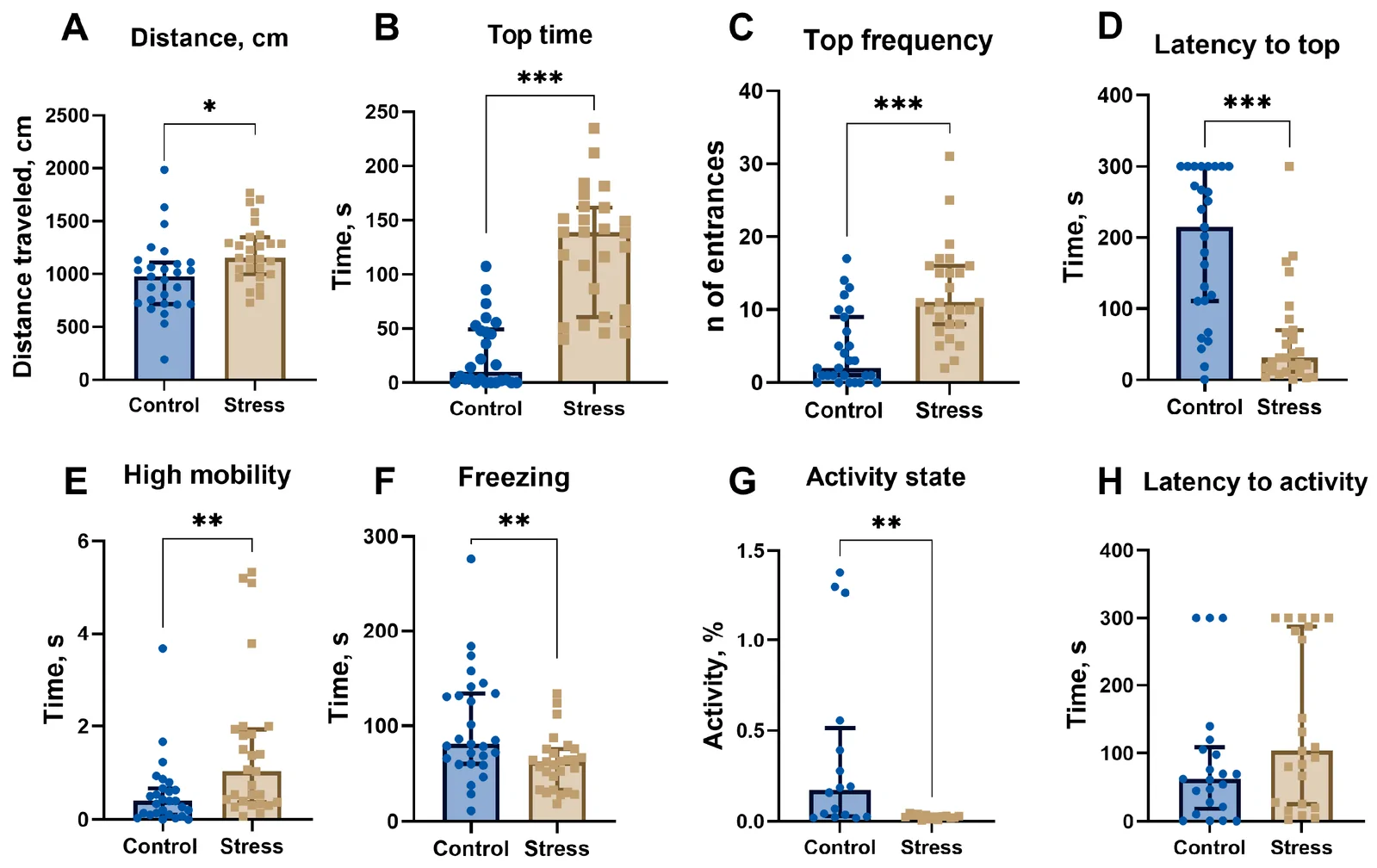
Effects of 2-week chronic unpredictable stress (CUS2) on adult zebrafish behavior (Experiment 1) in 5 min novel tank test (**A**–**F**) and zebrafish tail immobilization test (**G**,**H**). * *p* < 0.05, ** *p* < 0.01, *** *p* < 0.001, U-test. Data are presented as median ± interquartile range (n = 27 per group, with 1 outlier removed in control group for C and G, as well as 1 outlier removed in stress group for G; see Table 3 for statistical details).

**Figure 3:**
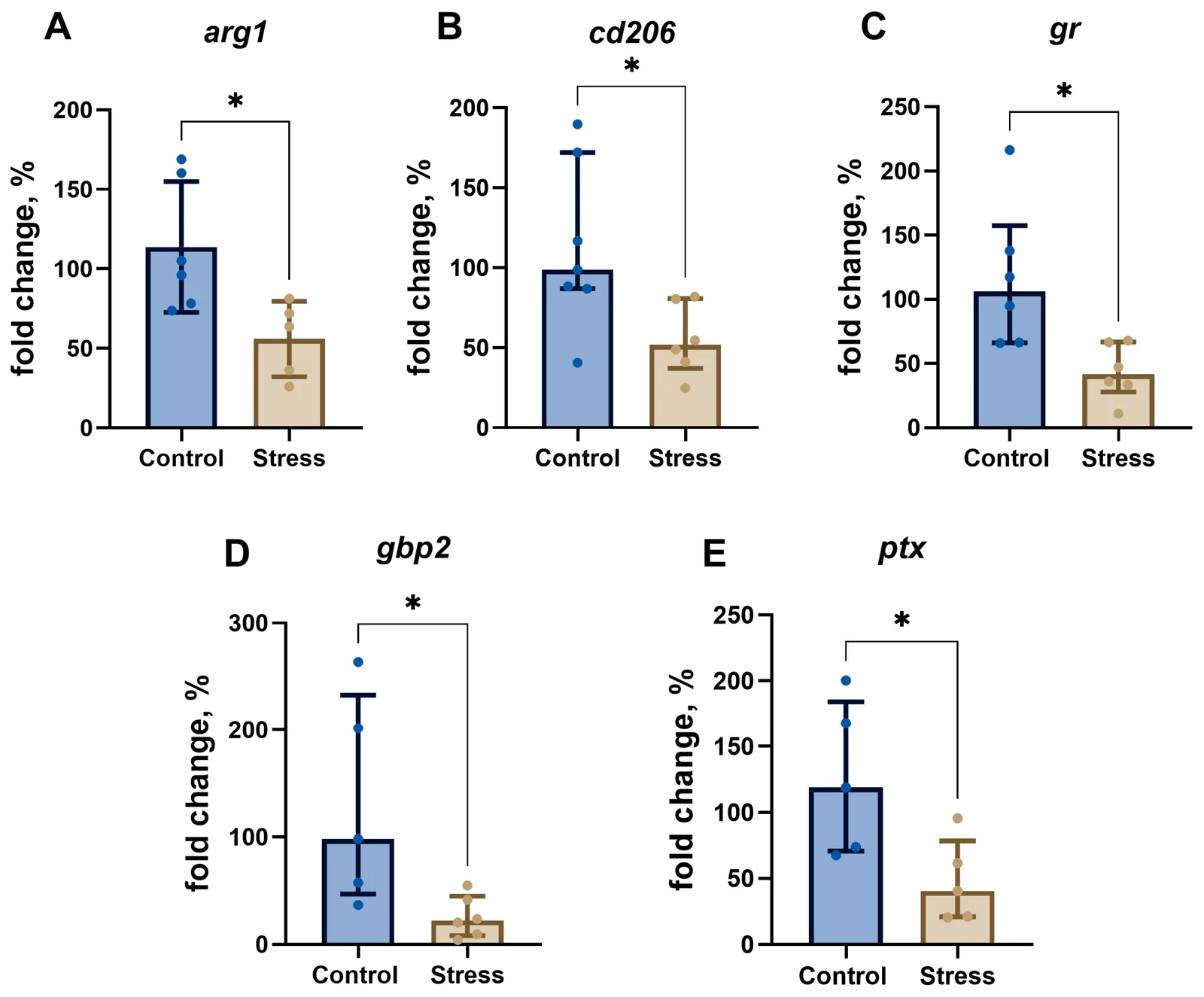
Effects of 2-week chronic unpredictable stress (CUS2) on brain expression of microglial (**A**,**B**), astroglial marker genes (**D**,**E**) and the glucocorticoid receptor gene (**C**) in adult zebrafish (Experiment 1), assessed by RT-PCR. * *p* < 0.05, U-test. Data are presented as median ± interquartile range (n = 6–7 per group without outliers, with 1 outlier removed in control for (**C**–**E**), 1 outlier removed in stress for (**B**–**E**), and 2 outliers in stress for (**A**), see Table 3 statistical details).

*Experiment 1 yielded a significant stress effect on anxiety-like behavior and neuroinflammatory gene expression (Table 3).* Specifically, CUS2-exposed zebrafish exhibited increased distance traveled, top time, frequency of top entries, and duration of high mobility, and also reduced latency to the top, NTT freezing duration and ZTI activity (Figure 2). CUS2 downregulated the expression of *gr*, two M2 microglial biomarker genes (*arg1*, *cd206*), and the A2 astroglial marker gene (*ptx*), but upregulated the A1 marker *gbp2* (Table 3 and Figure 3).

### 3.2. Effects of Minocycline and Nimesulide on Behavior

**Figure 4:**
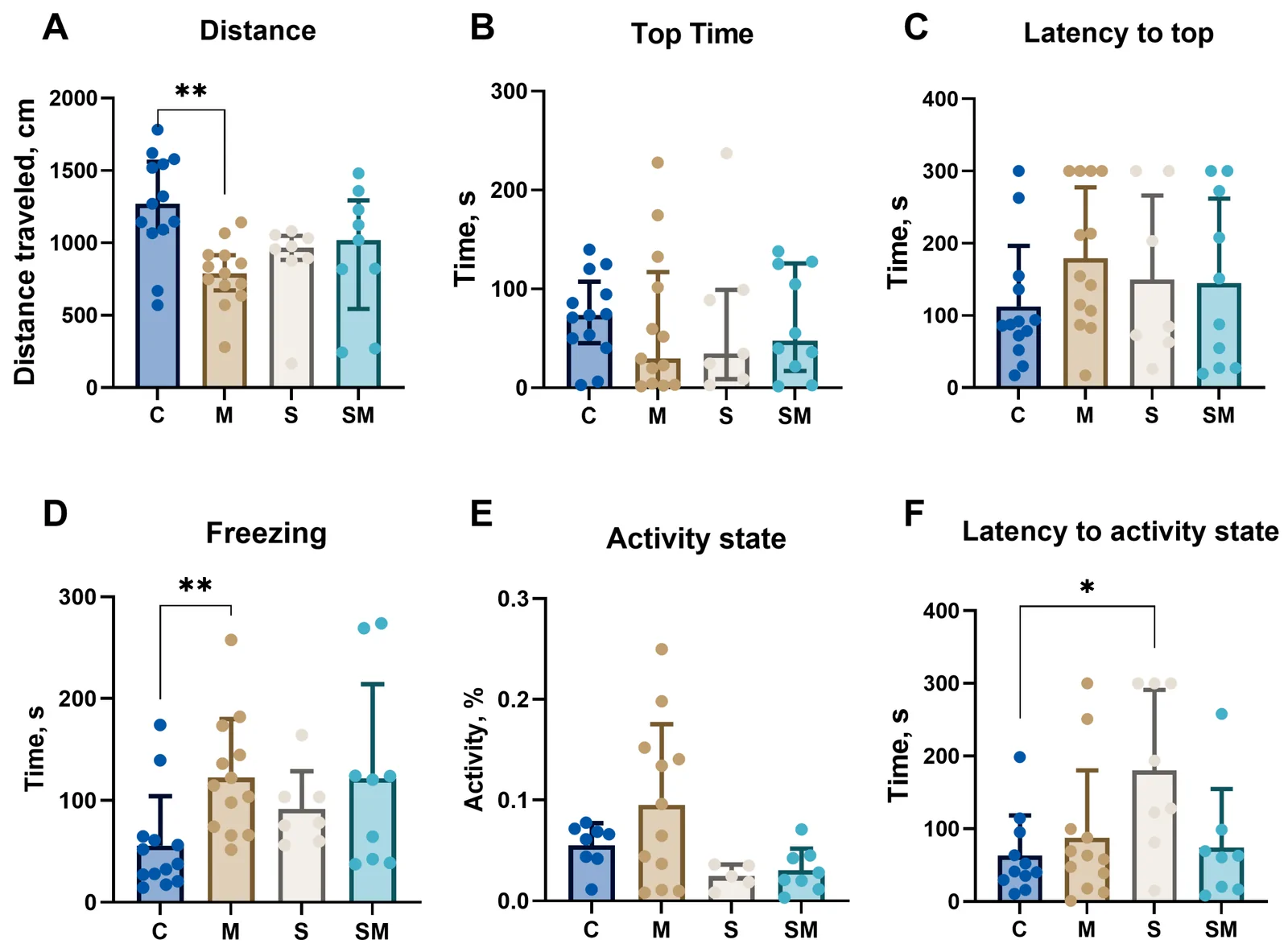
Effects of 2-week chronic unpredictable stress (CUS2) and minocycline treatment (50 mg/L for 2 week) on adult zebrafish behavior (Experiment 2) in the 5 min novel tank test (**A**–**D**) and zebrafish tail immobilization test (**E**,**F**). C—control group (n = 13), M—minocicline treatment (n = 13 for NTT, 10 for ZTI), S—CUS2 (n = 8 for NTT, 6–8 for ZTI), SM—stress group + minocycline treatment (n = 9 for NTT, 8 for ZTI). * *p* < 0.05, ** *p* < 0.01, post hoc Dunn test for significant Kruskal–Wallis test data. All data present as median ± interquartile range (n = 13–6 per group without outliers, with 2 outliers removed in control group for E, and 1 outlier removed for stress group for D and E, see Table 3 for statistical details).

Experiment 2 showed a significant minocycline treatment effect on hypoactivity/sedative- and depression-like behavior and neuroinflammatory gene expression (Table 4), as the drug at 50 mg/L (given for 2 weeks) reduced the distance traveled and increased freezing duration in the NTT vs. the unstressed control group. In the ZTI test, the latency to move increased in the CUS2, but not the CUS2+minocycline groups (Table 4, Figure 4).

**Figure 5:**
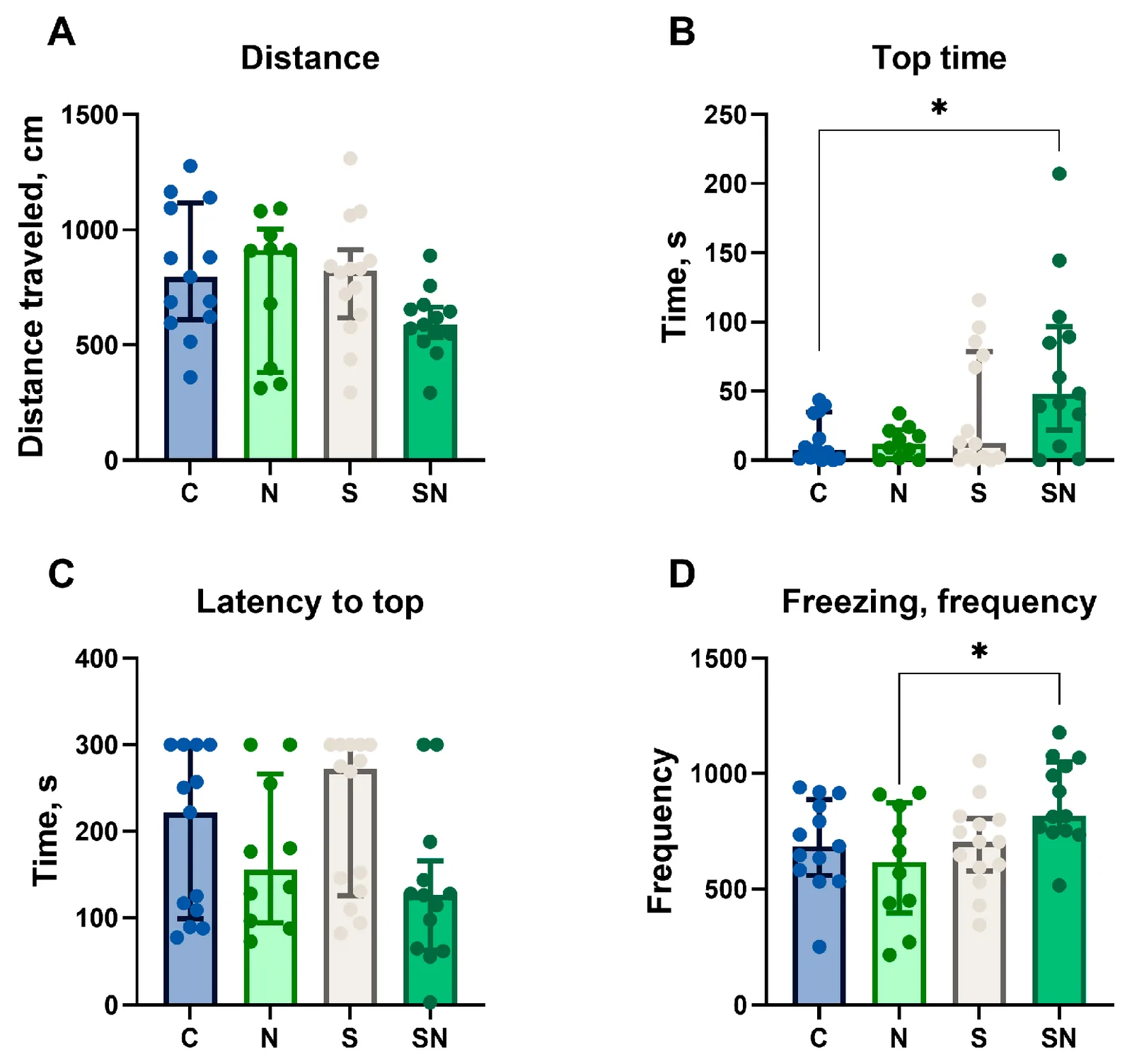
Effects of 2-week chronic unpredictable stress (CUS2) and nimesulide treatment (2.5 mg/L for 1 week) on adult zebrafish behavior (Experiment 3) in the 5 min novel tank test (**A**–**D**). C—control (n = 13), N—nimesulide treatment (n = 10), S—CUS2 (n = 14), SN—CUS2+nimesulide treatment (n = 13). * *p* < 0.05, post hoc Dunn test for significant Kruskal–Wallis test data. All data present as median ± interquartile range (n = 10–14 per group; all data included, see Table 5 for statistical details).

Experiment 3 revealed significant nimesulide treatment effects on anxiety-like behavior and neuroinflammatory gene expression (Table 5). Adding nimesulide during stress increased top time compared to the control group and freezing duration compared to the nimesulide-alone group (Table 5, Figure 5).

**Table 4:** Analysis of deviance (Type III, Wald test) for Experiment 2 (see Figure 4 and Figure 6). NS—no significant data (*p* > 0.05), df = 1, Chisq—wald Chi-square statistic, Pr (>Chisq)—probability value (*p*-value), where Pr < 0.05 indicates statistical significance.

Parameters	Factors	Chisq	Pr (>Chisq)
*The novel tank test (NTT)*			
Distance, cm	Stress	1.221	0.269, NS
	Treatment	4.140	0.042
	Stress × Treatment	6.296	0.012
Top time, sec	Stress	0.000	1.000, NS
	Treatment	0.122	0.727, NS
	Stress × Treatment	0.005	0.946, NS
Top frequency, n	Stress	0.074	0.785, NS
	Treatment	0.593	0.441, NS
	Stress × Treatment	4.058	0.044
Latency to top, s	Stress	0.027	0.869, NS
	Treatment	0.923	0.337, NS
	Stress × Treatment	1.247	0.264, NS
Duration of high mobility, s	Stress	1.546	0.214, NS
	Treatment	0.878	0.349, NS
	Stress × Treatment	8.450	0.004
Duration of freezing, s	Stress	6.371	0.012
	Treatment	17.354	<0.001
	Stress × Treatment	6.542	0.011
Frequency of freezing	Stress	0.133	0.715, NS
	Treatment	1.221	0.269, NS
	Stress × Treatment	8.631	0.003
*The zebrafish tail immobilization (ZTI) test*			
Activity, %	Stress	9.519	0.002
	Treatment	0.399	0.528, NS
	Stress × Treatment	0.390	0.532, NS
Latency to activity state, sec	Stress	2.088	0.148, NS
	Treatment	0.856	0.355, NS
	Stress × Treatment	3.868	0.049
*Gene expression analyses*			
*nos2a*	Stress	12.209	<0.001
	Treatment	12.498	<0.001
	Stress × Treatment	7.177	0.007
*cox2*	Stress	7.626	0.006
	Treatment	5.149	0.023
	Stress × Treatment	0.674	0.412, NS
*il1β*	Stress	0.152	0.697, NS
	Treatment	0.592	0.442, NS
	Stress × Treatment	0.000	0.992, NS
*il6*	Stress	13.520	<0.001
	Treatment	1.122	0.290, NS
	Stress × Treatment	1.747	0.186, NS
*arg1*	Stress	9.674	0.002
	Treatment	10.296	0.001
	Stress × Treatment	6.730	0.009
*cd206*	Stress	2.179	0.140, NS
	Treatment	3.857	0.050
	Stress × Treatment	1.768	0.184, NS
*c3*	Stress	0.538	0.463, NS
	Treatment	1.568	0.211, NS
	Stress × Treatment	0.126	0.723, NS
*gbp2*	Stress	1.161	0.281, NS
	Treatment	0.323	0.570, NS
	Stress × Treatment	0.151	0.697, NS
*s100a10*	Stress	12.102	<0.001
	Treatment	0.057	0.811, NS
	Stress × Treatment	14.467	<0.001
*Ptx*	Stress	8.541	0.003
	Treatment	2.959	0.085, NS
	Stress × Treatment	2.656	0.103, NS

**Figure 6:**
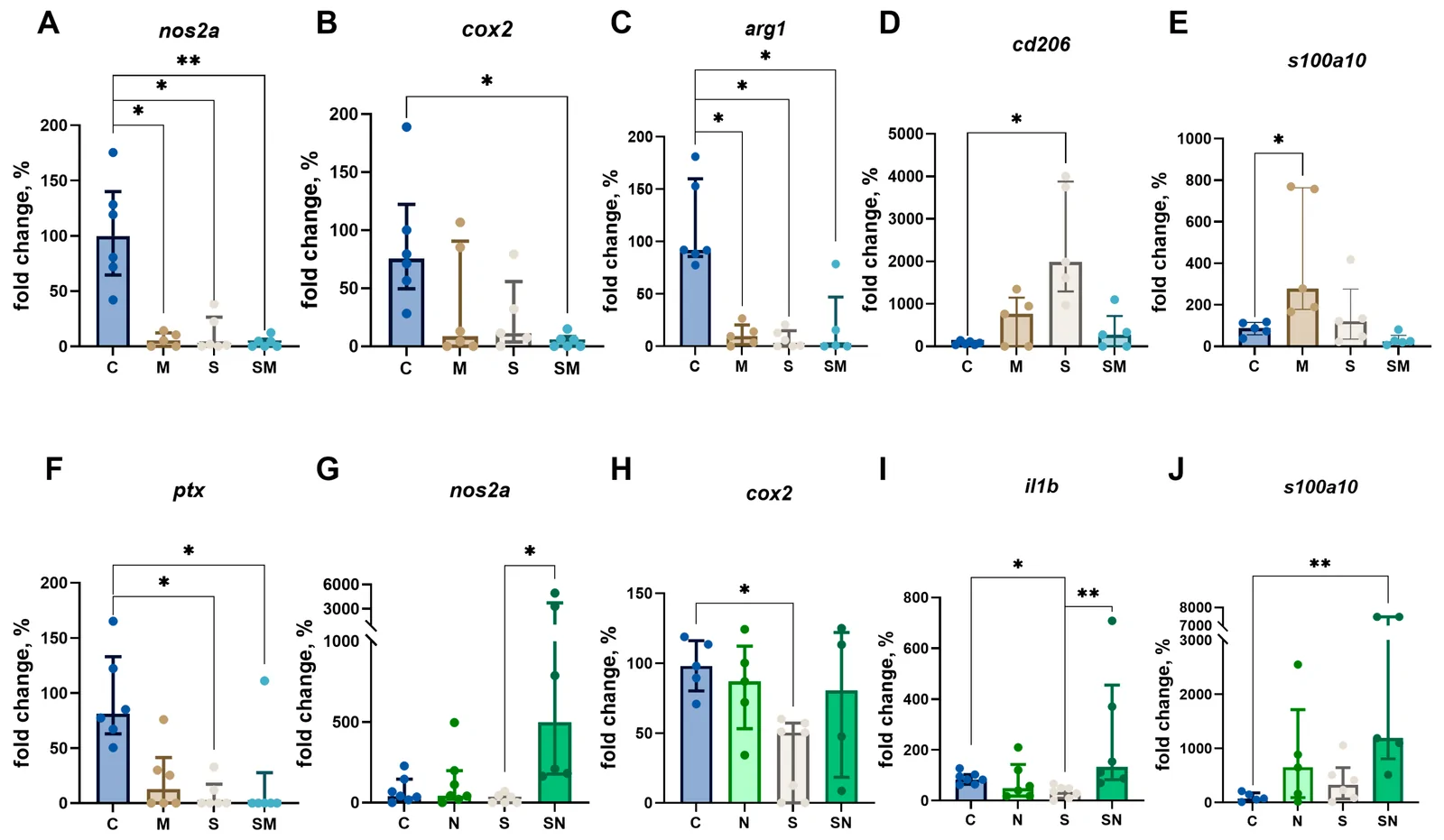
Effects of 2-week chronic unpredictable stress (CUS2) and anti-inflammation drug administration (minocycline at 50 mg/L for 2 weeks and nimesulide 2.5 mg/L for 1 week) on brain expression of microglial (**A**–**D**,**F**–**I**) and astroglial (**E**,**J**) neuroinflammation biomarker genes in adult zebrafish (Experiments 2 and 3), assessed by RT-PCR. C—control (n = 6 for Experiment 2, 7 for Experiment 3), M –minocicline treatment (n = 6), S—CUS2 (n = 6 for Experiment 2, 7 for experiment 3), SM—CUS2+minocycline treatment (n = 6), N—nimesulide treatment (n = 7), SN—CUS2+nimesulide treatment (n = 7). * *p* < 0.05, ** *p* < 0.01, post hoc Dunn test for significant Kruskal–Wallis test data. Data are presented as median ± interquartile range (n = 6–7 per group, with 1 outlier removed in C for H, 1 outlier removed in M for A and F, 1 outlier removed in S for A, D, E and G, 1 outlier removed in SM for E, 1 outlier removed in N for I and J, 2 outliers removed in N for H, 1 outlier removed in SN for G and I, and 2 outliers removed in SN for H and J, see Table 4 and Table 5 for statistical details).

**Table 5:** Analysis of deviance (Type III, Wald test) for Experiment 3 (see Figure 5 and Figure 6). NS—no significant data (*p* > 0.05), df = 1. Chisq—wald Chi-square statistic, Pr (>Chisq)—probability value (*p*-value), where Pr < 0.05 indicates statistical significance.

Parameters	Factors	Chisq	Pr (>Chisq)
*The novel tank test (NTT)*			
Distance, cm	Stress	1.872	0.171, NS
	Treatment	3.090	0.079, NS
	Stress × Treatment	0.798	0.372, NS
Top time, sec	Stress	10.788	0.001
	Treatment	1.663	0.197, NS
	Stress × Treatment	2.180	0.140, NS
Top frequency, n	Stress	0.000	0.990, NS
	Treatment	2.335	0.126, NS
	Stress × Treatment	4.608	0.032
Latency to top, s	Stress	0.315	0.575, NS
	Treatment	4.295	0.038
	Stress × Treatment	1.643	0.200, NS
Duration of high mobility, s	Stress	1.076	0.300, NS
	Treatment	2.162	0.141, NS
	Stress × Treatment	1.777	0.183, NS
Duration of freezing, s	Stress	1.852	0.174, NS
	Treatment	1.075	0.300, NS
	Stress × Treatment	0.547	0.459, NS
Frequency of freezing	Stress	4.798	0.028
	Treatment	0.355	0.552, NS
	Stress × Treatment	5.044	0.025
*The zebrafish tail immobilization (ZTI) test*			
Activity, %	Stress	0.892	0.345, NS
	Treatment	0.043	0.835, NS
	Stress × Treatment	6.019	0.014
Latency to activity state, sec	Stress	2.945	0.086, NS
	Treatment	0.417	0.518, NS
	Stress × Treatment	7.669	0.006
*Gene expression analyses*			
*nos2a*	Stress	1.087	0.297, NS
	Treatment	3.883	0.049
	Stress × Treatment	5.841	0.016
*cox2*	Stress	4.346	0.037
	Treatment	0.861	0.353, NS
	Stress × Treatment	1.764	0.184, NS
*il1β*	Stress	0.079	0.778, NS
	Treatment	4.220	0.040
	Stress × Treatment	10.824	0.001
*il6*	Stress	0.509	0.476, NS
	Treatment	1.868	0.172, NS
	Stress × Treatment	0.477	0.490, NS
*arg1*	Stress	0.155	0.694. NS
	Treatment	0.121	0.727, NS
	Stress × Treatment	0.010	0.919, NS
*cd206*	Stress	0.388	0.533, NS
	Treatment	4.186	0.041
	Stress × Treatment	1.462	0.227, NS
*c3*	Stress	1.522	0.217, NS
	Treatment	0.701	0.402, NS
	Stress × Treatment	6.872	0.009
*gbp2*	Stress	0.467	0.494, NS
	Treatment	0.389	0.533, NS
	Stress × Treatment	1.233	0.267, NS
*s100a10*	Stress	4.498	0.034
	Treatment	9.015	0.003
	Stress × Treatment	0.341	0.559, NS
*Ptx*	Stress	2.178	0.140, NS
	Treatment	1.322	0.250, NS
	Stress × Treatment	0.758	0.384, NS

### 3.3. Transcriptional Effects of Minocycline and Nimesulide

The observed behavioral changes following CUS2 were accompanied by altered neurotranscriptomic responses as well. In Experiment 2, CUS2 downregulated the expression of an M1 marker *nos2a*, M2 marker *arg1*, and A2 marker *ptx*, but upregulated another M2 marker, *cd206*, vs. control. Minocycline alone similarly downregulated *nos2a* and *arg1* while upregulating the A2 marker *s100a10*. When administered during CUS2, minocycline downregulated the expression of M1 markers *nos2a* and *cox2* and M2 marker *arg1* vs. control and normalized the expression level of the A2 marker *ptx* (Table 4 and Figure 6). In Experiment 3, CUS2 downregulated the expression of an M1 marker *cox2* and the inflammatory cytokine *il1β*, whereas the combined CUS2+nimesulide group showed upregulated expression of *nos2a* (vs. CUS2), *s100a10* (vs. control), and normalized the expression of *cox2* and *il1β* vs. control (Table 5 and Figure 6).

## 4. Discussion

The present study is the first report examining CUS2 effects on zebrafish behavior and its pharmacological modulation by two distinct anti-inflammatory drugs, minocycline and nimesulide. Overall, the CUS2 exposure alone induced despair/depression-like behavior in the ZTI test without inducing concurrent anxiety-like responses in the NTT. At the molecular level, this behavioral profile was accompanied by the downregulation of brain expression of *gr* and anti-inflammatory glial markers (*cd206*, *arg1*, *ptx3*), without affecting pro-inflammatory genetic biomarkers. In contrast, chronic treatment with minocycline (50 mg/L) effectively reversed stress-induced depression-like behavior, whereas nimesulide failed to produce behavioral effects at non-toxic concentrations tested.

As already noted, reduced ZTI activity (indicative of despair/depression-like behavior) was also evoked by CUS2 here. Interestingly, however, this affective phenotype was not accompanied by increased anxiety-like NTT behavior—a pattern that differs from the comorbid anxiety/depression-like phenotype often reported in zebrafish after longer-term chronic stress protocols [53,56]. While increased NTT time in top and shorter latency to zone can formally be interpreted as anxiolytic-like effect, it cannot be excluded that the observed behavioral profile partially reflects heightened exploratory activity (characteristic of the ‘resistance’ phase of stress), during which the organism mobilizes adaptive mechanisms to compensate for adverse conditions [70]. Another alternative explanation can be that increased top behavior reflects natural ethological exploratory activity in response to a novel, quiet environment, rather than a simple disruption of habituation. For example, since control (unstressed) fish also experienced an undisturbed environment in the days preceding testing, the heightened exploration in the CUS2 (vs. control) group may represent altered, rather than merely reduced, anxiety-like response. Furthermore, because bottom dwelling represents a natural antipredatory response and a primary safe zone in zebrafish, the observed increase in both the frequency and duration of top entries can also reflect a disruption of this innate protective behavior. As such, the observed shift away from the bottom zone may be driven by stress-induced behavioral disinhibition, hyperactivity, or an altered exploratory drive, highlighting a complex alteration in the fish’s natural risk-assessment mechanisms rather than a straightforward anxiolytic effect (e.g., [70] see for discussion).

Nevertheless, several lines of indirect evidence suggest that this response cannot be considered a true ‘anxiolysis’, since early manifestations of affective pathogenesis were already evident. For example, increased locomotor activity in fish was accompanied by prolonged episodes of high mobility, suggesting erratic (chaotic) swimming (a well-established sign of stress and anxiety in fish [44,71]). In contrast, anxiolysis would typically be accompanied by a more structured and orderly exploration. Likewise, the combination of a hyperactive NTT profile with the decreased ZTI activity, suggestive of higher behavioral despair, conflicts with a low-stress/anxiety phenotype in CUS2-exposed fish. At the molecular level, CUS2 downregulated *gr* expression, suggesting the onset of glucocorticoid resistance rather than a successful stress adaptation and/or a calming effect. Thus, the behavioral profile observed in the NTT may potentially reflect a putative transitional state bridging active stress resistance and the onset of fully developed affective pathogenesis.

Indeed, this specific behavioral profile (depression-like behavior without overt anxiety) may be tentatively interpreted within the framework of a putative *transitional* stress phase, wherein animals might have progressed beyond initial stress resistance but have not yet developed the full ‘combined’ spectrum of pathological responses associated with chronic stress exposure. Neurobiologically, this phase may represent a critical window where early molecular stress markers (e.g., *gr* downregulation and altered glial functional state biomarkers) emerge prior to the activation of major neuroinflammatory cascades. From a pathogenic perspective, this hypothesized transitional state may reflect a period of heightened vulnerability, where therapeutic interventions could potentially prevent the progression to established affective disorders before comorbid symptoms and key neuropathological changes consolidate.

In general, this idea is consistent with dynamic temporal patterns in stress-induced CNS alterations documented across multiple species. For example, in both rodents and zebrafish, one-week CUS rarely yields robust or consistent behavioral or molecular changes [56,72], suggesting some initial period of stress resistance. In contrast, CUS2 exposure marks the onset of detectable pathological signs in rodents and zebrafish, including behavioral alterations (primarily depression-like responses) that emerge with early molecular shifts (including gr downregulation and reduced anti-inflammatory glial marker expression), yet without the fully blown neuroinflammatory cascades [55,73,74]. At the same time, longer (e.g., 5- or 12-week) zebrafish CUS protocols do show comorbid NTT anxiety-like and ZTI depression-like phenotypes, accompanied by pronounced neuroinflammatory markers (elevated pro-inflammatory cytokines, M1 microglial polarization) and neuroendocrine alterations (sustained cortisol dysregulation) [56]. Taken together, these observations suggest the CUS2 model used here is likely an intermediate time point in stress pathogenesis, where initial depression-like symptoms and molecular stress biomarkers begin to emerge, but the fully blown ‘comorbid’ affective behavioral phenotype and major established neuroinflammation characteristic of chronic stress have not yet developed.

From a conceptual standpoint, the possibility that CUS2 may represent a transitional stress phase could also potentially offer a framework for better understanding and reconciling conflicting data from various studies utilizing CUS2 protocols in both rodent and zebrafish models. Indeed, the literature diverges on whether this duration induces anxiety-like behavior, as some reports note prominent anxiogenic effects in rodents and fish [55,73,74], while others demonstrate more complex outcomes. For instance, a rat CUS2 protocol induces transient anxiolytic-like, followed by delayed anxiogenic-like, responses [75]. Likewise, 2-week mild CUS in rodents may also fail to alter anxiety-like behavior while still inducing depression-like states (e.g., see [76]). In zebrafish, however, systematic comparisons of temporal stress dynamics remain limited. The present study provides some evidence that CUS2 in fish can produce depression-like phenotypes without overt anxiety (also see rodent models, where chronic social defeat stress induces a temporal progression from initial anxiety-like to later depression-like states [77,78]).

In the present study, CUS2 primarily induced depression-like behavior in zebrafish, accompanied by downregulated *gr* expression—the molecular alteration that parallels stress responses at early time points in both rodent and zebrafish models [55,79], where behavioral and neuroendocrine changes emerge before the pronounced neuroinflammation. This pattern is also in line with the view on CUS2 as a putative transitional affective phase, as discussed above, and raises the possibility that zebrafish (like rodents [77,78]) may indeed differentially display some anxiety- and depression-like responses during CUS, rather than develop a ‘combined’ affective syndrome, as noted in [48]. Furthermore, while our PCR analyses revealed downregulation of glial biomarker genes associated with anti-inflammatory phenotypes (*cd206*, *arg1*, *ptx3*), this occurred without concurrent upregulation of pro-inflammatory biomarkers (*nos2a*, *cox2*), suggesting that overt neuroinflammation was likely not yet present at this time point. Since M2-like microglia and astroglia provide essential trophic support to neurons and facilitate neurogenesis [80,81], their compromised function suggests impaired neural plasticity [82,83] that, in turn, may further contribute to the emergence of depression-like ZTI behavior. As already noted, linking CUS2 to a putative transitional stress phase may help reconcile seemingly contradictory rodent findings, where some 2-week stress studies report anxiety-like behavior [55,73,74] while others observe primarily depression-like responses [75,76].

In line with this notion, administering 50 mg/L minocycline throughout the CUS2 protocol did not alter anxiety-like NTT behavior, but reduced ZTI test hypoactivity, effectively reversing the despair-like phenotype. These results are generally consistent with reported antidepressant-like properties of minocycline well-documented in both rodent and zebrafish models [35,84,85,86] and in clinical studies [87], which is largely attributed to the suppression of microglia-driven neuroinflammation (as shown both in vitro [35,88] and in vivo [85,89,90,91,92]). A key mechanism of this activity likely involves the inhibition by the drug of pro-inflammatory M1-associated microglial responses and their phagocytic activity [93]. On the one hand, our data corroborate this profile by showing downregulated M1-associated markers (*nos2a*, *cox2*). On the other hand, minocycline effects on anti-inflammatory M2-like phenotype remain poorly understood. While rodent models of amyotrophic lateral sclerosis and in vitro models report that M1 suppression can be seen without M2 modulation [89], other studies indicate M2 polarization of microglia in rat models of intracerebral hemorrhage and ischemia–reperfusion rat models [92,93], in line with our zebrafish findings of downregulated M2-associated *arg1*. Taken together, this suggests that minocycline can improve CUS2-evoked behavior and suppress M1 biomarkers without concurrent upregulation of M2 markers. However, it remains to be tested whether this reflects a specific feature of CUS2 (e.g., in which neuroinflammatory cascades are not yet fully established within a 2-week stress), the drug-specific preferential targeting of M1 pathways at this concentration, putative species-specific differences in microglial polarization dynamics between zebrafish and rodents, or alternative non-inflammatory mechanisms of minocycline action in the brain (e.g., direct modulation of monoaminergic transmission, neuronal plasticity or epigenetic modulation). Given the chronic nature of CUS used here, testing the latter possibility, including epigenetic modulation of both neuronal and glial cells by both drugs tested, merits further in-depth scrutiny.

Furthermore, anti-inflammatory CNS effects of minocycline appear to be dose-dependent in various model systems [84,86,89,90,91,92]. For instance, in a mouse alcohol-induced depression model, the drug alleviates anhedonia at 30 mg/kg, but additionally reduces despair-like behavior at 50 mg/kg when administered for 2 weeks [86]. This finding partly informed our selection of 50 mg/L for the present CUS2 study, as higher doses may be required to achieve robust antidepressant-like effects in chronic stress paradigms. However, we also noted that 50 mg/L concentration produced a modest reduction in baseline locomotor activity, which may reflect mild sedation. Therefore, future studies employing lower, behaviorally inert concentrations (e.g., 25 mg/L) and their comprehensive molecular profiling may provide a more interpretable test to definitively disentangle motor vs. affective drug effects. Minocycline action on astroglia must also be considered, because pro-inflammatory microglia can activate neurotoxic A1-like astrocytic responses [94]. In line with this notion, rodent methamphetamine abstinence and chronic post-surgical pain stress models show that minocycline may indeed suppress A1-like activation while promoting protective A2-associated astroglial function [95,96]. Although these findings originate from non-psychological stress pathology models, they demonstrate the capacity of minocycline to modulate microglia–astrocyte crosstalk. Our observation of increased A2-associated *s100a10* expression indirectly supports a potential ‘dual’ modulation of astrocytes by minocycline.

Interestingly, in our pilot experiments, minocycline given at 25 mg/L during CUS2 produced a weaker anxiolytic-like effect than a conventional serotonergic anxiolytic/antidepressant drug fluoxetine at 0.1 mg/L (Supplementary Materials Table S2 and Figure S3), suggesting that targeting microglia-driven inflammation alone may be insufficient to achieve therapeutic anxiolytic effects of selective serotonin reuptake inhibitors (SSRIs). Although minocycline has both antidepressant-like and anti-neuroinflammatory effects in the zebrafish CUS5 model [85], the CUS2 employed here did not evoke overt upregulation of CNS pro-inflammatory markers (indicative of neuroinflammation). However, because minocycline still produced positive behavioral effects in this model even in the absence of pronounced neuroinflammation, it is possible that behavioral profile of minocycline may involve mechanisms beyond microglial suppression per se, such as direct modulation of neurotransmission, epigenetic regulation and/or neuronal plasticity pathways, clearly meriting further analyses.

The present study was also the first evaluation of nimesulide effects on anxiety- and depression-like behavior in zebrafish, failing to produce significant effects on them. Prior research on this and other NSAIDs documents both anxiolytic and antidepressant efficacy clinically [14,97] and in various rodent depression models [98,99,100], including CUS [101,102]. However, direct dose comparisons are limited by differences in administration routes (oral/injection in rodents vs. immersion in zebrafish). Nevertheless, the concentrations safe for zebrafish were likely insufficient to replicate the efficacy seen in rodent studies, which utilized longer treatment durations (up to 3 weeks). In our pilot experiments, nimesulide at 5 mg/L, as well as at 2.5 mg/L during CUS2, administered for 2 weeks, resulted in considerable fish mortality (Supplementary Materials Figure S2), likely due to its known hepato- and mitochondrial toxicity [103], hence raising concerns regarding the safety of its prolonged use in affective disorders.

At the molecular level, the 1-week nimesulide treatment during CUS2 induced a complex response in glial-associated markers, with the upregulation of *nos2a* and *s100a10*, but normalized stress-altered *cox2* and *il1β* expression. However, despite these molecular alterations, nimesulide did not produce overt behavioral effects in the present study. This dissociation between molecular and behavioral outcomes indicates that while the drug engages with glial targets, the current dosing regimen, likely constrained by safety risks, may not be sufficient to link such molecular changes to behavioral recovery. Thus, further analyses using a broader range of safe concentrations and/or extended treatment durations may be necessary to assess pharmacological mechanisms and behavioral efficacy of nimesulide in zebrafish CUS models.

Moreover, this study has several other limitations. First, the putative ‘transitional stress phase’ proposed here remains an interpretative framework, and while our data are compatible with this notion, definitive confirmation necessitates longitudinal studies tracking behavioral and molecular trajectories across multiple time points of CUS. Although we maintained an approximately 1:1 male-to-female ratio across experimental groups, sex was not included as a variable in our analyses. As such, subdividing groups and evaluating sex-dependent responses in future studies with larger CUS and drug-treated cohorts would be valuable. Likewise, for molecular analyses, we utilized the M1/M2 and A1/A2 classification framework based on the established use of these genes as classical, evolutionarily conserved glial markers in both rodent and zebrafish studies. However, because whole-brain mRNA expression cannot definitively establish cellular-specific polarization, future studies employing histological approaches, such as immunohistochemistry or single-cell RNA sequencing, are needed to confirm cellular localization and validate these phenotypic states more fully. While the current CUS2 protocol was designed to induce robust changes within a short timeframe, the combination of stressors used here was considerable. Future research can therefore benefit from exploring milder, more ecologically valid and less variable stress paradigms (e.g., chronic social stress models already being developed for adult zebrafish [104,105]). Testing other zebrafish strains (e.g., AB, TU, WK), as well as age groups (e.g., juvenile and old fish), as well as focusing on stratification of zebrafish cohorts into stress-prone and stress-resistant, may also be interesting in the context of the present study. Collectively, this can provide a model with higher translational validity, more closely resembling the chronic, low-grade stress often experienced in human conditions in various clinical populations.

Moreover, neuroinflammatory biomarkers were assayed in the whole-brain samples, and did not assess potential region-specific patterns of glial activation. Future studies employing immunohistochemistry or region-specific molecular analyses may help delineate neuroanatomical correlates of stress and treatment effects examined here. Additional measures of CUS effects, such as cortisol levels and brain monoamines, may be useful. Our study also focused exclusively on central neuroinflammatory markers and did not assess systemic inflammatory responses. Chronic stress is known to evoke both peripheral and central inflammatory cascades, and these processes may interact bidirectionally via the gut–brain axis, vagal signaling, or circulating cytokines. Thus, as we did not measure peripheral inflammatory biomarkers (e.g., plasma cytokines, splenic immune cell profiles), we cannot determine that CNS effects of minocycline and nimesulide observed here were mediated solely by central mechanisms, peripheral mechanisms, or their interaction. As such, future studies incorporating parallel assessment of systemic and neuroinflammatory markers will be needed to dissect these contributions further.

Finally, our study focused primarily on key inflammatory markers of microglia and astroglia in the brain. However, because behavioral effects of minocycline and nimesulide were observed even in the apparent absence of pronounced neuroinflammation here, other mechanisms of their effects may also be involved, in addition to anti-inflammatory. Thus, expanding the range of analyzed biomarkers, particularly those associated with the diverse, non-immune functions of glia, can therefore be useful. For example, mounting evidence links specialized microglial phenotypes, such as those expressing the triggering receptor expressed on myeloid cells 2 (TREM2), in synaptic regulation and neurodegeneration [106]. As minocycline modulates TREM2 expression in microglial cells in arsenic-exposed rodent models [90,107,108], this may represent another putative pathway contributing to neuroactive effects of the drug, independent of canonical anti-inflammatory action.

## 5. Conclusions

In summary, our study demonstrates that CUS2 in zebrafish can induce despair/depression-like behavior without necessarily worsening anxiety-like responses and neuroinflammation markers. This behavioral and molecular profile is compatible with the interpretation of CUS2 as a putative early transitional phase of stress pathogenesis, distinct from the ‘affective syndrome’ with comorbid anxiety–depression phenotype (typically observed in fish after longer stress exposure [55]). Such a temporal window is interesting and, if it exists, may offer an opportunity for pharmacological intervention before the consolidation of full affective pathology. Minocycline (50 mg/L) effectively reversed stress-induced depression-like behavior, whereas nimesulide failed to produce behavioral effects at non-toxic concentrations, despite inducing a complex response in glial-associated markers, highlighting the need for further targeted research to fully elucidate its pharmacological profile. The lack of nimesulide’s efficacy here may reflect its narrow therapeutic window in zebrafish, as higher doses resulted in considerable mortality (likely due to hepato- and mitochondrial toxicity), suggesting that drug-specific safety profiles, rather than anti-inflammatory mechanisms per se, determine therapeutic feasibility in vivo.

Moreover, the CUS2 model applied here was characterized by distinct molecular alterations (e.g., *gr* and anti-inflammatory glial marker downregulation) and behavioral phenotypes (depression-, but not anxiety-like responses) than longer (e.g., CUS5 or more) stress that does involve neuroinflammation with comorbid anxiety+depression-like symptoms (e.g., [58,86]). Thus, CNS treatment efficacy may depend on the timing of intervention during the disease progression—the conclusion found in fish that may translate into human therapy. Furthermore, glial experimental modulation by anti-inflammatory drugs here produced behavioral effects even in the absence of neuroinflammation biomarker changes, suggesting that early CUS-related behavioral deficits may involve additional, non-canonical inflammatory cascades beyond those targeted by minocycline and nimesulide. Future studies may involve probing putative additional CNS effects of glial-modulating drugs, comparing shorter (e.g., CUS2) vs. prolonged (e.g., CUS5+) stress paradigms, as well as incorporating measures of systemic inflammation, to more fully delineate the role of neuroimmune mechanisms across the dynamic progression of affective disorders.

## Figures and Tables

**Table 1:** Protocol of the 2-week chronic unpredictable stress (CUS2) used in the present study, according to [54], with minor modifications (see Figure 1 for the study design).

Day 1—Bright light (1500 lux) for 8 h + cold exposure (10 °C) for 24 h + single exposure to alarm pheromone exposure (5 mL of alarm pheromone extracted from additional intact fish) Day 2—Shallow water (30% of maximum, vol/vol) for 24 h + vortexing (10 fish/50 mL test tube at 1000 rpm) for 30 s Day 3—Net chasing in home tank for 20 min + three 1-min electric shocks (0.1 V/cm) + food deprivation for 24 h Day 4—Multicolored light-emitting diode (LED) strips 10 cm away from bottom and around the home tank (a total of 300 red, green, blue and white lights—activated in a randomized stroboscopic mode; color change frequency: 0.5–2 Hz, light intensity ~300 lux near the tank walls) for 8 h + shallow water for 24 h Day 5—Social isolation for 24 h (placing individual fish in 90 mL plastic cups) + food deprivation for 24 h Day 6—Cold exposure + three 1-min air exposures (lifting fish out of the tank by a net and leaving them in the air) + vortexing for 30 s Day 7—Exposure to a different zebrafish strain (10 Glofish) for 8 h + shallow water for 24 h + three 1-min electric shocks Day 8—Hyperthermia (32 °C) for 8 h + net chasing in home tank for 20 min + three 1-min electric shocks Day 9—Crowding/novelty stress in green/blue/red 8-L plastic bucket for 8 h (10 fish/L) + three 1-min electric shocks Day 10—Social isolation for 24 h + food deprivation for 24 h Day 11—Cold exposure + vortexing for 30 s + alarm pheromone exposure in the home tank Day 12—Bright light for 8 h + hyperthermia (32 °C) for 8 h Day 13—Multicolored lights around the home tank for 24 h + net chasing in home tank for 20 min Day 14—Hyperthermia (28 °C) for 8 h + three 1-min electric shocks + three 1-min air exposures Day 15—Behavior testing in the novel tank test (NTT) and zebrafish tail immobilization (ZTI) test Day 16—Euthanasia + brain collection

**Table 2:** List of biomarker gene primers used in the study.

Genes	Forward	Reverse	Product Length
*M1 pro-inflammatory microglia*			
*nos2a*	TCAGGTACGGTGTCTTTGGC	CTCTTCCTGATCCGCCACTG	520
*cox2*	TTCTTC CAGCATTTCTCTCAC	TTTGTGTTGAACCTCCAGCGTCTC	132
*il1β*	TTCCCCAAGTGCTGCTTATT	AAGTTAAAACCGCTGTGGTCA	149
*il6*	TCAACTTCTCCAGCGTGATG	TCTTTCCCTCTTTTCCTCCTG	308
*M2 anti-inflammatory microglia*			
*arg1*	TCCGTTCTCCAAAGGACAGC	GACTCGTCGTTGGGAAGGTT	150
*cd206*	CGACACAGATGGCAGATGGAAGAC	ACGCTTCTTTGACTCAGGACAGTTC	131
*A1 pro-inflammatory astroglia*			
*c3*	TGATTCTGGCTCGCAGTGATGATG	CATGGCTGAGGCTGGACAGTTATC	134
*gbp2*	GGTCCTGGAGGCCAAACTCA	AGCCCGGCAAAAACAACGTG	191
*A2 anti-inflammatory astroglia*			
*s100a10*	CTGGATGCCAATGGAGACGG	ATCCACCCTGTAGCAAGCCT	96
*ptx3*	AGCATCCGGGAACAGCTGAG	AAGCAATCCATGGGGCGAGA	135
*Housekeeping genes*			
*act-b*	CATCAGGGTGTCATGGTTGGT	TCTCTTGCTCTGAGCCTCATCA	69
*rpl13*	AGTAGTCAGGTGTCCGACCA	GTGCGGTATTCCTTCAGCCT	
*Glucocorticoid receptor (stress biomarker)*			
*gr*	ACAGCTTCTTCCAGCCTCAG	CCGGTGTTCTCCTGTTTGAT	116

**Table 3:** Summary of U-test results for Experiment 1 (see Figure 2 and Figure 3). NS—no significant data (*p* > 0.05).

Parameters	Me (Control)	Me (Stress)	U, *p*
*The novel tank test (NTT)*			
Distance, cm	978	1152	190, *p* = 0.0022
Top time, sec	14	139	74, *p* < 0.0001
Top frequency, n	2	11	120, *p* < 0.0001
Latency to top, s	215	31	110, *p* < 0.0001
Duration of high mobility, s	0.4	1.03	192, *p* = 0.0023
Duration of freezing, s	81	63	193, *p* = 0.0026
Frequency of freezing	616	604	358, *p* = 0.91
*The zebrafish tail immobilization (ZTI) test*			
Activity, %	0.17	0.03	40, *p* = 0.0021
Latency to activity state, sec	41	29	186, *p* = 0.12 NS
*Gene expression analyses*			
*PCR*			
*nos2a*	75	116	17, *p* = 0.94 NS
*cox2*	93	72	16, *p* = 0.88 NS
*il1β*	111	93	15, *p* = 0.75 NS
*il6*	77	92	23, *p* = 0.90 NS
*arg1*	101	64	2, *p* = 0.02
*cd206*	99	52	5, *p* = 0.04
*c3*	86	173	16, *p* = 0.32 NS
*gbp2*	98	22	2, *p* = 0.02
*s100a10*	46	200	12, *p* = 0.13 NS
*Ptx*	119	41	2, *p* = 0.03
*Gr*	106	42	4, *p* = 0.03

## Data Availability

The data that support the findings of this study are available upon a reasonable collaborative request from the corresponding author.

## Supplementary Materials

The following supporting information can be downloaded at https://www.mdpi.com/article/10.3390/brainsci16080815/s1. Supplementary Figure S1. Effects of minocycline (top row) and nimesulide (bottom row) on zebrafish behavior in the 5-min novel tank test. Minocycline: control (n = 14), 25 mg/L (n = 10), 50 mg/L (n = 10), 100 mg/L (n = 10). Nimesulide: control (n = 11), 1 mg/L (n = 10), 5 mg/L (n = 10). * *p* < 0.05, post-hoc Dunn’s test for significant Kruskal–Wallis data (see Supplementary Table S1 for details). Data are presented as median ± interquartile range; Supplementary Figure S2. Effects of nimesulide exposure on animal mortality during 2-week chronic unpredictable stress (CUS2) protocol in adult zebrafish (A 5 mg/L, B—2.5 mg/L) of during only second week of UCS (C, 2.5 mg/L). C—control, N—nimesulide, S—CUS2, SN—CUS2+nimesulide. Data are presented as percentage mortality per group (n = 15 per group); Supplementary Figure S3. Effects of 2-week chronic unpredictable stress (CUS2) and 1-week drug administration (minocycline 25 mg/L or fluoxetine 0.1 mg/L) on adult zebrafish behavior in the 5-min novel tank test (A–D) and 5-min zebrafish tail immobilization test (E,F). Groups: C—control, F—1-week fluoxetine (0.1 mg/L), M—1-week minocycline (25 mg/L), S—CUS2, SF—CUS2+fluoxetine, SM—CUS2+minocycline. * *p* < 0.05, ** *p* < 0.01, **** *p* < 0.0001, post-hoc Dunn’s test for significant Kruskal–Wallis test data (see Supplementary Table S2 for details). Data are presented as median ± interquartile range (n = 13–15 per group); Supplementary Figure S4. Effects of 2-week chronic unpredictable stress (CUS2) on brain expression of microglial (A,B) and astroglial biomarker genes (D,E) and the glucocorticoid receptor gene (*gr*) in adult zebrafish (Experiment 1). Microglial-specific biomarker genes included *arg1* (arginase-1) and *cd206* (mannose receptor C type 1). Astroglial-specific biomarker genes included *gbp2* (guanylate-binding protein 2) and *ptx* (pentraxin 3). * *p* < 0.05, U-test. Data are presented as median ± interquartile range (n = 6–7 per group, all data was analyzed without removing the outliers (red dots), also see Supplementary Table S3 for statistical details); Supplementary Figure S5. Effects of 2-week chronic unpredictable stress (CUS2) and anti-inflammation drug administration (minocycline at 50 mg/L for 2 weeks and nimesulide 2.5 mg/L for 1 week) on brain expression of microglial (A–D, F–I), astroglial (E,J) and neurounlammation marker genes in adult zebrafish (Experiment 2, 3), assessed by RT-PCR. Microglial-specific biomarker genes included *nos2a* (nitric oxide synthase 2a), *cox2* (cyclooxygenase-2), *arg1* (arginase-1), *cd206* (mannose receptor C type 1) and *il1β* (interleukin 1β). Astroglial-specific biomarker genes included *c3* (complement component 3), *gbp2* (guanylate-binding protein 2), *s100a10* (s100 calcium binding protein a10) and *ptx* (pentraxin 3). Groups: C—control (n = 6 for Experiment 2, 7 for Experiment 3), M—minocycline (n = 6), S—CUS2 (n = 6 for Experiment 2, 7 for Experiment 3), SM—CUS2+minocycline (n = 6), N –nimesulide (n = 7), SN—CUS2+nimesulide (n = 7). * *p* < 0.05, ** *p* < 0.01, post-hoc Dunn test for significant Kruskal–Wallis test data. Data are presented as median ± interquartile range (data was analyzed without removal of the outliers (red dots), see Supplementary Tables S4 and S5 for statistical details); Supplementary Table S1. Summary of Kruskal–Wallis (KW) test results for various drug concentrations in the 5-min novel tank test (see Supplementary Figure S1 for data). Me (group)—median. NS—no significant data (*p* > 0.05); Supplementary Table S2. Summary of Kruskal–Wallis (KW) test results for fluoxetine (0.1 mg/L) and minocycline (25 mg/L) following the CUS2 protocol in the 5-min novel tank test (NTT) and zebrafish tail immobilization test (ZTI) (see Supplementary Figure S3 for details). Me (group)—median. NS—no significant data (*p* > 0.05); Supplementary Table S3. Summary of U-test results for gene expression analyzes in Experiment 1 (Supplementary Figure S4). Me (group)—median. NS—no significant data (*p* > 0.05). All data was analyzed without removing the outliers; Supplementary Table S4. Analysis of deviance (Type III, Wald test) for Experiment 2 (Supplementary Figure S5). NS—no significant data (*p* > 0.05), df = 1. All data was analyzed without removing the outliers. Chisq—wald Chi-square statistic, Pr (>Chisq)—probability value (*p*-value), where Pr < 0.05 indicates statistical significance; Supplementary Table S5. Analysis of deviance (Type III, Wald test) for Experiment 3 (Supplementary Figure S5). NS—no significant data (*p* > 0.05), df = 1. All data was analyzed including outliers. Chisq—wald Chi-square statistic, Pr (>Chisq)—probability value (*p*-value), where Pr < 0.05 indicates statistical significance.

## Abbreviations

The following abbreviations are used in this manuscript:

ACT-β	Beta-actin
ARG1	Arginase-1
C3	Complement component 3
CD206	Mannose receptor C type 1
CNS	Central nervous system
COX2	Cyclooxygenase-2
CUS	Chronic unpredictable stress
DMSO	Dimethyl sulfoxide
GBP2	Guanylate-binding protein 2
GR	Glucocorticoid receptor
IL1β	Interleukin 1β
IL6	Interleukin 6
NOS2A	Nitric oxide synthase 2a
NSAIDs	Non-steroidal anti-inflammatory drugs
NTT	Novel tank test
PTX3	Pentraxin 3
RPL13	Ribosomal protein l13
S100A10	S100 calcium binding protein a10
SSRIs	Selective serotonin reuptake inhibitors
TREM2	Triggering receptor expressed on myeloid cells 2
ZTI	Zebrafish tail immobilization (test)
