# Supplementary material for: Neurobehavioral Effects of Two Distinct Anti-Inflammatory Drugs in a Two-Week Chronic Stress Model in Zebrafish
Source: Brain Sci. 2026 Jul 31;16(8):815. doi: 10.3390/brainsci16080815 (PMC13510721; doi:10.3390/brainsci16080815)
Supplement: Supplementary file 1 [file brainsci-16-00815-s001.zip › brainsci-4413894-supplementary.pdf]

Supplementary Materials

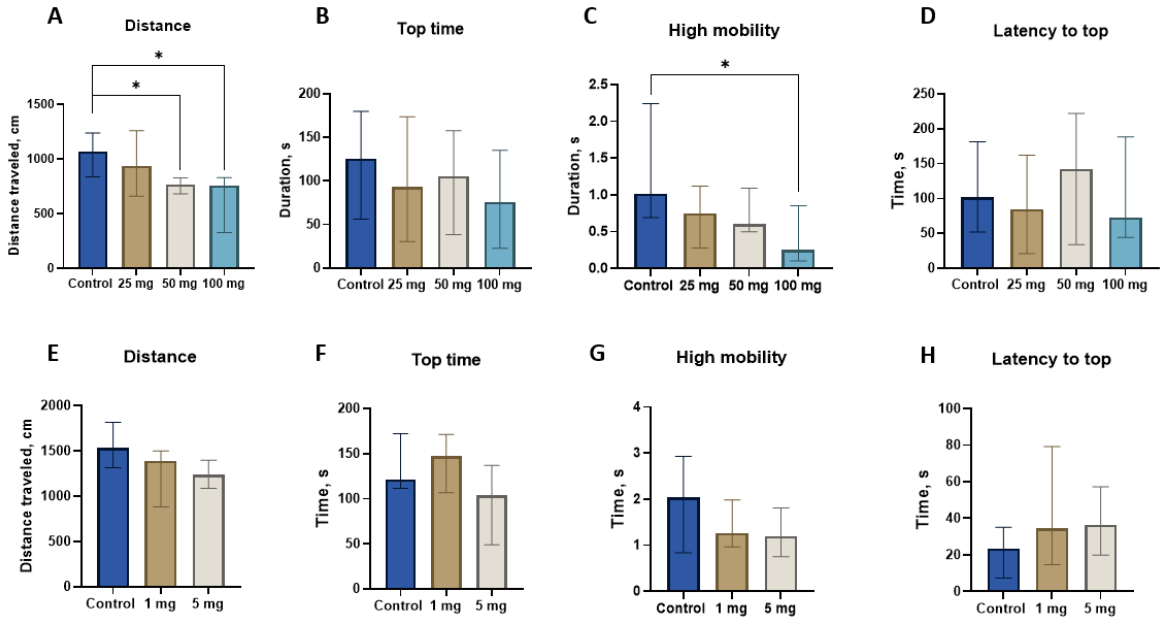

Supplementary Figure S1. Effects of minocycline (top row) and nimesulide (bottom row) on zebrafish behavior in the 5-min novel tank test. Minocycline: control (n = 14), 25 mg/l (n = 10), 50 mg/l (n = 10), 100 mg/l (n = 10). Nimesulide: control (n=11), 1 mg/l (n =10), 5 mg/l (n = 10). *$p < 0.05$, post-hoc Dunn's test for significant Kruskal–Wallis data (see Supplementary Table 1S for details). Data are presented as median ± interquartile range.

Supplementary Table S1. Summary of Kruskal–Wallis (KW) test results for various drug concentrations in the 5-min novel tank test (see Supplementary Fig. 1S for data). Me(group) – median. NS – no significant data (p>0.05).

Parameters	Me(c)	Me(25)	Me(50)	Me(100)	KW $H_{(3, 43)}$, P
Minocycline					
Distance, cm	1070	937	764	760	13.03, p = 0.0046
Top time, sec	126	93	106	76	1.54, p = 0.67 NS
Top frequency, n	11	7	5	9	3.23, p = 0.36 NS
Latency to top, s	102	84	142	73	0.59, p = 0.89 NS
Duration of high mobility, s	1	0.75	0.6	0.25	8.54, p = 0.036
Duration of freezing, s	94	97	144	134	9.33, p = 0.025
Frequency of freezing	560	521	559	824	2.12, p = 0.55 NS
Nimesulide					
		**Me(c)**	**Me(1)**	**Me(5)**	**$H_{(2, 31)}$, P**
Distance, cm		1537	1492	1275	1.41, p = 0.49 NS
Top time, sec		122	147	104	3.17, p = 0.21 NS
Top frequency, n		16	19	12	5.59, p = 0.06 NS
Latency to top, s		24	35	37	2.83, p = 0.24 NS
Duration of high mobility, s		2	1.3	1.2	2.72, p = 0.26 NS
Duration of freezing, s		24	23	40	1.65, p = 0.44 NS
Frequency of freezing		388	402	479	0.36, p = 0.84 NS

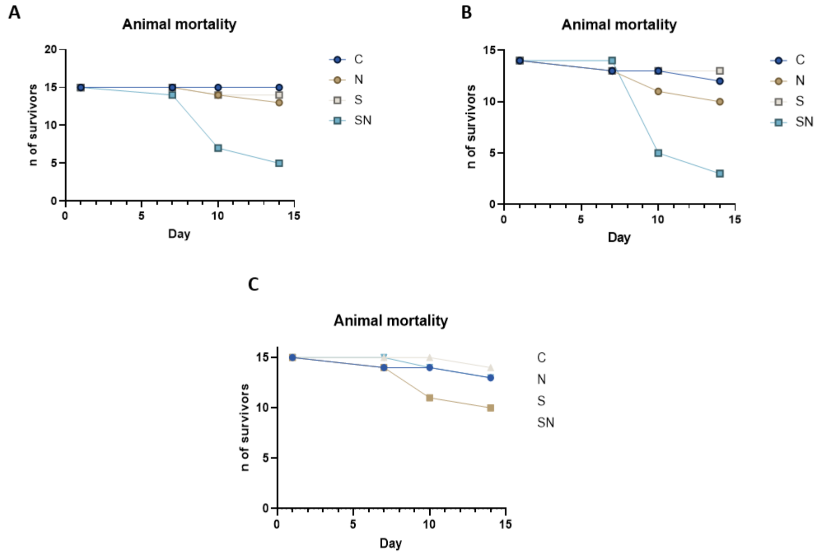

Supplementary Figure S2. Effects of nimesulide exposure on animal mortality during 2-week chronic unpredictable stress (CUS2) protocol in adult zebrafish (A 5 mg/L, B - 2.5 mg/L) of during only second week of UCS (C, 2.5 mg/L). C – control, N – nimesulide, S – CUS2, SN – CUS2 + nimesulide. Data are presented as percentage mortality per group (n = 15 per group).

A separate experiment examined the effects of chronic 1-week minocycline (25 mg/l) and fluoxetine (0.1 mg/l) administration during CUS2 on anxiety- and depression-like behaviors (n =15 per group). Fluoxetine (Biocom Ltd., Stavropol, Russia) was administered at 0.1 mg/L starting from day 8 of CUS2 protocol. The fluoxetine group and the UCS + fluoxetine group both received 1 mL of DMSO, to control for potential solvent effects.

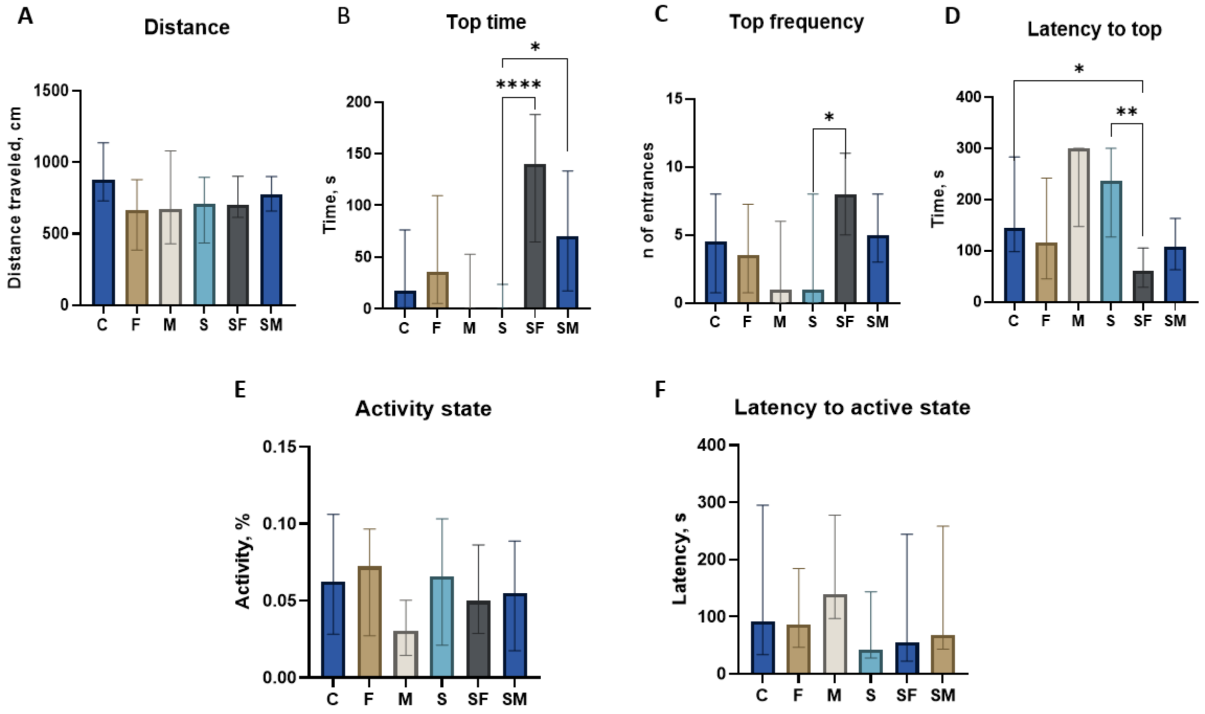

Supplementary Figure S3. Effects of 2-week chronic unpredictable stress (CUS2) and 1-week drug administration (minocycline 25 mg/l or fluoxetine 0.1 mg/l) on adult zebrafish behavior in the 5-min novel tank test (A-D) and 5-min zebrafish tail immobilization test (E, F). Groups: C- control, F – 1-week fluoxetine (0.1 mg/l), M – 1-week minocycline (25 mg/l), S – CUS2, SF – CUS2+fluoxetine, SM – CUS2+minocycline. *p < 0.05, **p < 0.01, ****p < 0.0001, post-hoc Dunn's test for significant Kruskal–Wallis test data (see Supplementary Table 2S for details). Data are presented as median ± interquartile range (n = 13-15 per group).

Supplementary Table S2. Summary of Kruskal–Wallis (KW) test results for fluoxetine (0.1 mg/L) and minocycline (25 mg/L) following the CUS2 protocol in the 5-min novel tank test (NTT) and zebrafish tail immobilization test (ZTI) (see Supplementary Fig. 3S for details). Me(group) – median. NS – no significant data (p>0.05).

**Parameters**	**Me(c)**	**Me(f)**	**Me(m)**	**Me(s)**	**Me(sf)**	**Me(sm)**	**KW H, P**
The novel tank test							$\mathbf{H_{(5, 87), P}}$
Distance, cm	881	664	674	712	701	775	8.86, p = 0.114 NS
Top time, sec	18	36	1	1	139	70	27.69, p<0.0001
Top frequency, n	5	4	1	1	8	5	13.29, p = 0.0208
Latency to top, s	144	116	300	237	61	108	21.36, p = 0.0007
Duration of high mobility, s	0.7	0.7	1.3	0.4	0.5	1	3.745, p = 0.58 NS
Duration of freezing, s	97	164	144	123	136	125	8.16, p = 0.15 NS
Frequency of freezing	569	601	482	622	883	762	35.41, p <0.0001
ZTI test							
Activity, %	0.06	0.07	0.03	0.06	0.05	0.05	6.53, p = 0.26 NS
Latency to active state, sec	92	87	140	43	55	68	5.89, p = 0.32 NS

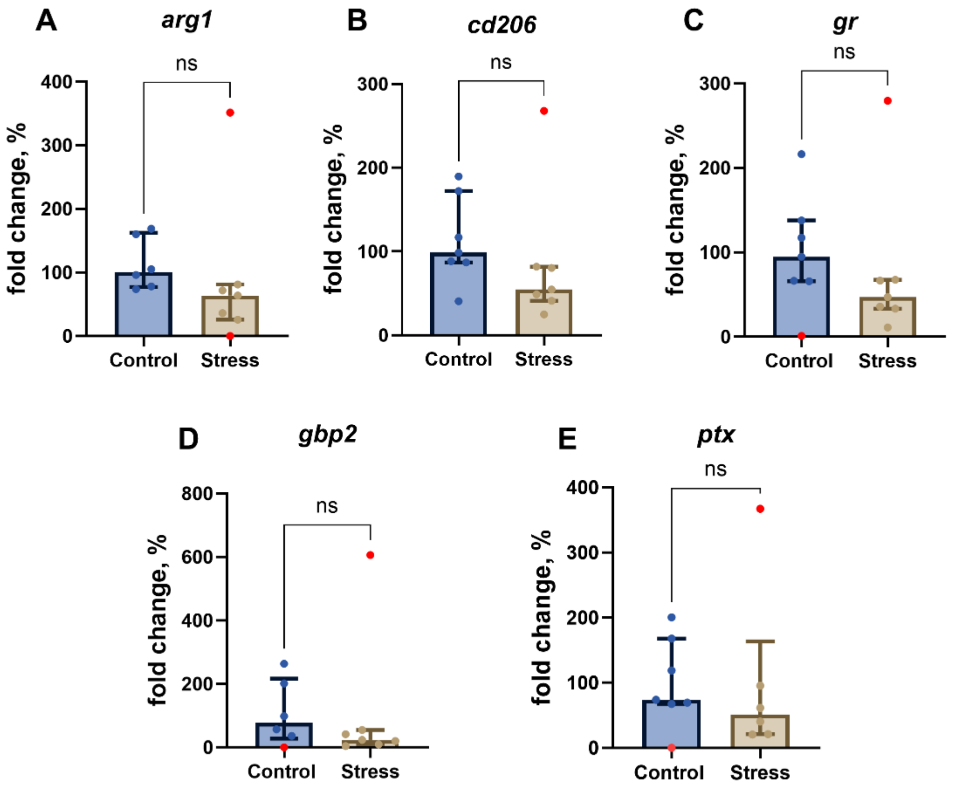

Supplementary Figure S4. Effects of 2-week chronic unpredictable stress (CUS2) on brain expression of microglial (A, B) and astroglial biomarker genes (D, E) and the glucocorticoid receptor gene (gr) in adult zebrafish (Experiment 1). Microglial-specific biomarker genes included *arg1* (arginase-1) and *cd206* (mannose receptor C type 1). Astroglial-specific biomarker genes included *gbp2* (guanylate-binding protein 2) and *ptx* (pentraxin 3). *p<0.05, U-test. Data are presented as median ± interquartile range (n = 6 – 7 per group, all data was analyzed without removing the outliers (red dots), also see Supplementary Table 3S for statistical details).

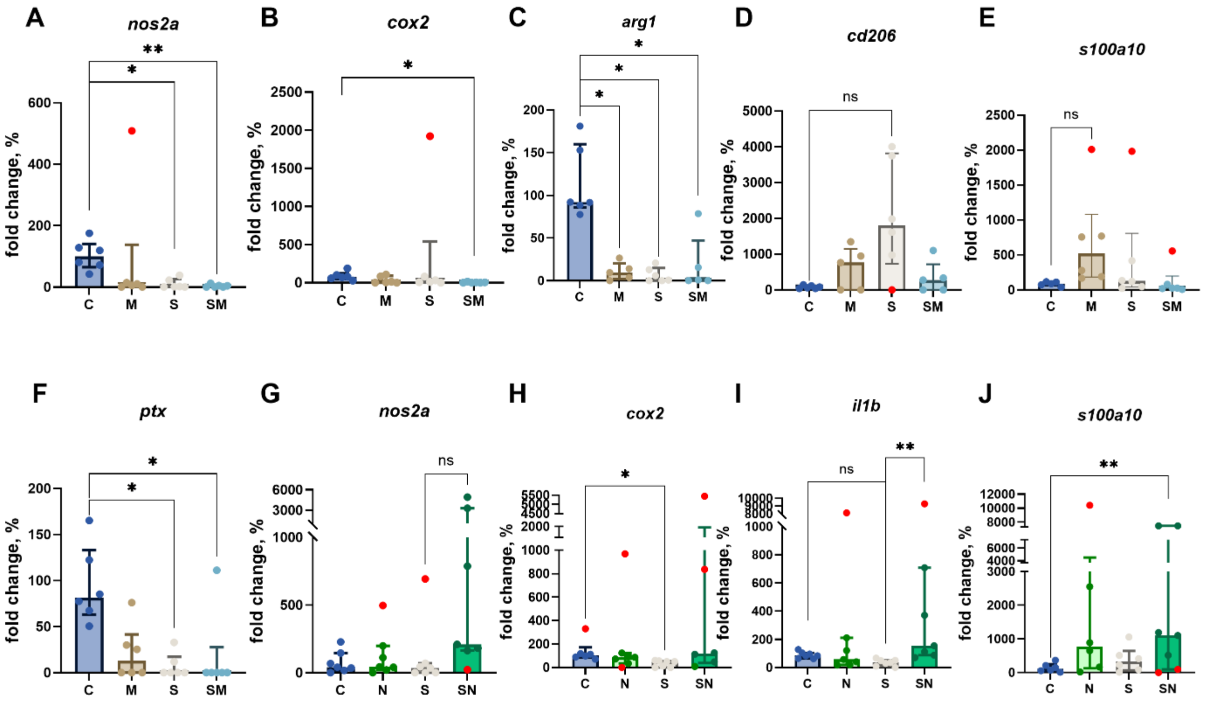

Supplementary Figure S5. Effects of 2-week chronic unpredictable stress (CUS2) and anti-inflammation drug administration (minocycline at 50 mg/l for 2 weeks and nimesulide 2.5 mg/l for 1 week) on brain expression of microglial (A-D, F-I), astroglial (E, J) and neurounlammation marker genes in adult zebrafish (Experiment 2, 3), assessed by RT-PCR. Microglial-specific biomarker genes included *nos2a* (nitric oxide synthase 2a), *cox2* (cyclooxygenase-2), *arg1* (arginase-1), *cd206* (mannose receptor C type 1) and *il1β* (interleukin 1β). Astroglial-specific biomarker genes included *c3* (complement component 3), *gbp2* (guanylate-binding protein 2), *s100a10* (s100 calcium binding protein a10) and *ptx* (pentraxin 3). Groups: C – control (n = 6 for Experiment 2, 7 for Experiment 3), M – minocicline (n = 6), S – CUS2 (n = 6 for Experiment 2, 7 for Experiment 3), SM – CUS2+minocycline (n = 6), N –nimesulide (n = 7), SN – CUS2+nimesulide (n = 7). *p<0.05, **p<0.01, post-hoc Dunn test for significant Kruskal–Wallis test data. Data are presented as median ± interquartile range (data was analyzed without removal of the outliers (red dots), see Supplementary Tables 4S and 5S for statistical details).

Supplementary Table S3. Summary of U-test results for gene expression analyzes in Experiment 1 (Supplementary Fig. 4S). Me(group) – median. NS – no significant data (p>0.05). All data was analyzed without removing the outliers.

**Genes**	**Me(control)**	**Me(stress)**	**U, P**
nos2a	75	116	17, p = 0.94 NS
cox2	93	72	16, p = 0.88 NS
il1β	111	93	15, p = 0.75 NS
il6	77	92	23, p = 0.90 NS
arg1	101	64	8, p = 0.07 NS
cd206	99	55	12, p = 0.13, NS
c3	86	173	16, p = 0.32 NS
gbp2	78	24	14, p = 0.37, NS
s100a10	46	200	12, p = 0.13 NS
ptx	74	51	15, p = 0.44, NS
gr	95	47	17, p = 0.38, NS

Supplementary Table S4. Analysis of deviance (Type III, Wald test) for Experiment 2 (Supplementary Fig. 5S). NS – no significant data (p>0.05), df = 1. All data was analyzed without removing the outliers. Chisq - wald Chi-square statistic, Pr (> Chisq) - probability value (p-value), where Pr < 0.05 indicates statistical significance.

Genes	Factors	Chisq	Pr (> Chisq)
nos2a	Stress	11.852	<0.001
	Treatment	4.913	0.03
	Stress x Treatment	2.336	0.13, NS
cox2	Stress	2.120	0.15, NS
	Treatment	8.911	0.003
	Stress x Treatment	0.012	0.91, NS
il1β	Stress	0.152	0.69, NS
	Treatment	0.592	0.44, NS
	Stress x Treatment	0.000	0.99, NS
il6	Stress	13.520	<0.001
	Treatment	1.122	0.29, NS
	Stress x Treatment	1.747	0.19, NS
arg1	Stress	9.674	0.002
	Treatment	10.296	0.001
	Stress x Treatment	6.730	0.009
cd206	Stress	0.442	0.51, NS
	Treatment	1.271	0.26, NS
	Stress x Treatment	1.046	0.31, NS
c3	Stress	0.538	0.46, NS
	Treatment	1.568	0.21, NS
	Stress x Treatment	0.126	0.72, NS
gbp2	Stress	0.118	0.73, NS
	Treatment	0.290	0.59, NS
	Stress x Treatment	1.396	0.23, NS
s100a10	Stress	2.860	0.09, NS
	Treatment	0.175	0.67, NS
	Stress x Treatment	8.914	0.003
ptx	Stress	11.543	<0.001, NS
	Treatment	4.640	0.03, NS
	Stress x Treatment	3.287	0.07, NS

Supplementary Table S5. Analysis of deviance (Type III, Wald test) for Experiment 3 (Supplementary Fig. 5S). NS – no significant data (p>0.05), df = 1. All data was analyzed including outliers. Chisq - wald Chi-square statistic, Pr (> Chisq) - probability value (p-value), where Pr < 0.05 indicates statistical significance.

**Genes**	**Factors**	**Chisq**	**Pr (> Chisq)**
nos2a	Stress	0.597	0.44, NS
	Treatment	5.466	0.02
	Stress x Treatment	3.946	0.047
cox2	Stress	0.516	0.47, NS
	Treatment	1.602	0.21, NS
	Stress x Treatment	4.956	0.03
il1β	Stress	0.102	0.75, NS
	Treatment	6.192	0.01
	Stress x Treatment	4.387	0.04
il6	Stress	0.509	0.48, NS
	Treatment	1.868	0.17, NS
	Stress x Treatment	0.477	0.49, NS
arg1	Stress	0.155	0.69, NS
	Treatment	0.121	0.73, NS
	Stress x Treatment	0.010	0.92, NS
cd206	Stress	0.388	0.53, NS
	Treatment	4.186	0.04
	Stress x Treatment	1.462	0.23, NS
c3	Stress	1.522	0.22, NS
	Treatment	0.701	0.40, NS
	Stress x Treatment	6.872	0.009
gbp2	Stress	0.467	0.49, NS

	Treatment	0.389	0.53, NS
	Stress x Treatment	1.233	0.27, NS
s100a10	Stress	0.063	0.80, NS
	Treatment	2.420	0.12, NS
	Stress x Treatment	0.343	0.56, NS
ptx	Stress	2.178	0.14, NS
	Treatment	1.322	0.25, NS
	Stress x Treatment	0.758	0.38, NS
